# Supported self-management for all with musculoskeletal pain: an inclusive approach to intervention development: the EASIER study

**DOI:** 10.1186/s12891-023-06452-4

**Published:** 2023-06-10

**Authors:** Emma L. Healey, Martyn Lewis, Nadia Corp, Noureen A. Shivji, Danielle A. van der Windt, Opeyemi O. Babatunde, Joanna Simkins, Bernadette Bartlam, Gill Rowlands, Joanne Protheroe

**Affiliations:** 1 Primary Care Centre Versus Arthritis, School of Medicine, Keele University, Keele, Staffordshire, ST5 5BG UK; 2 Musculoskeletal Outpatient Physiotherapy, Princess Royal Hospital, Apley Castle, Telford, TF1 6TF UK; 3 Population Health Sciences Institute, Newcastle University, Newcastle Upon Tyne, NE2 4HH UK

**Keywords:** Musculoskeletal pain, Health literacy, Supported self-management, Logic model, Mixed-methods

## Abstract

**Background:**

Supported self-management interventions for patients with musculoskeletal (MSK) conditions may not adequately support those with limited health literacy, leading to inequalities in care and variable outcomes. The aim of this study was to develop a model for inclusive supported self-management intervention(s) for MSK pain that take account of health literacy.

**Methods:**

A mixed methods study with four work-packages was conducted: work package 1: secondary analysis of existing data to identify potential targets for intervention; work package 2: evidence synthesis to assess effective components of self-management interventions taking into account health literacy; work package 3: views of community members and healthcare professionals (HCPs) on essential components; work package 4: triangulation of findings and an online modified Delphi approach to reach consensus on key components of a logic model.

**Findings:**

Findings identified targets for intervention as self-efficacy, illness perceptions, and pain catastrophizing. A range of intervention components were identified (e.g. information in diverse formats offered at specific times, action planning and visual demonstrations of exercise). Support should be multi-professional using a combination of delivery modes (e.g. remote, face-to-face).

**Conclusions:**

This research has developed a patient-centred model for a multi-disciplinary, multi-modal approach to supported self-management for patients with MSK pain and varying levels of health literacy. The model is evidence-based and acceptable to both patients and HCPs, with potential for significant impact on the management of MSK pain and for improving patient health outcomes. Further work is needed to establish its efficacy.

**Supplementary Information:**

The online version contains supplementary material available at 10.1186/s12891-023-06452-4.

## Background

Musculoskeletal (MSK) conditions affect over 17 million people across the UK [1], and are a major cause of pain and disability worldwide, with substantial impacts on both quality of life and healthcare resource use [2]. The mainstay of NHS treatment for MSK pain is supported self-management. However, research evidence for the effectiveness of supported self-management programmes for MSK health has been, at best, mixed [3, 4]. A Cochrane review of self-management programmes for osteoarthritis concluded that these programmes conferred ‘little or no benefit’ and recommended that future intervention development should consider patient health literacy (HL) to explore issues of health equity [5].

HL refers to the personal characteristics and social resources needed for individuals and communities to access, understand, appraise and use information and services to make decisions about health [6]. Limited HL is an issue with high prevalence in the UK and internationally [7]. Recent studies have shown that 43% of the English population have limited HL [7, 8]. This figure rises to 61% when health information involves numeracy [7, 8].

Limited HL is associated with poorer health, greater illness and increased healthcare costs [9–11]. People with low socio-economic status or low levels of education are also more likely to have poorer HL [12, 13]. Such health inequity has been recognised by the World Health Organization, which has identified HL as a critical determinant of health; one that empowers individuals and enables their engagement in health. It is integral to people developing the skills, knowledge and self-efficacy over their lifetimes to meaningfully act on that knowledge, within their cultural and political contexts, so as to maintain good health [14].

Health services are increasingly delegating the work of managing MSK conditions to patients themselves [15], yet people with limited HL are less likely to be able to do this successfully. Specific disadvantages that people with limited HL experience when living with MSK pain are related to understanding complex information about the condition and managing complicated medication regimes. The well-documented issues of limited HL and medication management are particularly important in MSK pain, as many medications can have severe or even life-threatening complications if not correctly managed [16–18].

There is evidence that healthcare professionals (HCPs) find pain management in patients with limited HL challenging [19]. Furthermore, the disappointing results of self-management approaches for patients with MSK pain may be partly explained by limited HL, suggesting that this under-served group requires better support to successfully manage their condition [20].

In the light of such evidence around the importance of HL and its role in the effective management of MSK pain, there is a need to develop acceptable and useful patient support, educational materials and/or patient resources to better support both such patients and the HCPs working with them [19–22].

The aim of the EASIER (achEs And painS and lIving bEtteR) study was to develop a logic model to explain how a supported self-management intervention could address potential mediators, and lead to improved health outcomes for people who have MSK pain and varying levels of HL.

## Methods

**Fig. 1:**
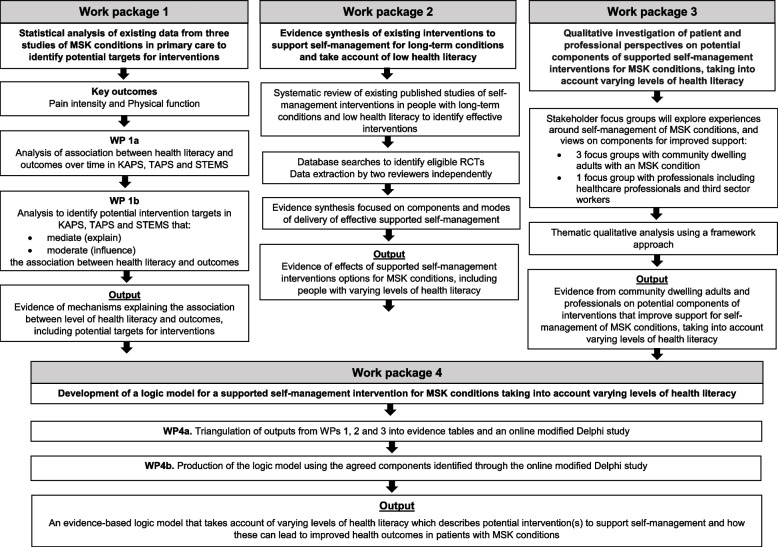
EASIER study work packages

The EASIER study consisted of 4 inter-linked work packages, in a mixed-methods concurrent-sequential design. Work Packages 1, 2 and 3 were conducted concurrently, and the findings were synthesized in work package 4 to produce an evidence-based logic model (see Fig. 1).

### Work Package 1 (WP1): Analysis of the relationship between HL and MSK health outcomes

The objective of WP1 was to use longitudinal analysis methods to investigate the relationship between limited HL and poor MSK health outcomes in existing datasets and identify potential intervention target(s) by examining factors that influence (moderate) and explain (mediate) the relationship.

The study team took a pragmatic approach to selecting the datasets for analysis in work package 1. The studies chosen were readily available internal datasets that members of the EASIER study team had been involved in previously and were familiar with. We analysed data from three existing studies (two prospective cohort designs, one cluster randomized controlled trial (RCT)) including patients presenting with MSK pain in primary care (Keele Aches and Pains Study (KAPS) [23], Treatment of Aches and PainS (TAPS) [24] and Stepping up the Evidence for Musculoskeletal Services (STEMS) [25] to further investigate the effect of limited HL on MSK health outcomes in three different samples. KAPS, TAPS and STEMS provide rich sources of data (including socio-demographic, psychological and consultation-related factors), which provided the opportunity to examine the underlying mechanisms of the relationship between HL and musculoskeletal pain and function outcomes (see Additional file 1).

#### Sample size calculation

The sample size requirement was based on expected power of 0.8, with up to 7 observed variables on expected direct effects (0.1 to 0.45) for a structural equation model (mediation and moderation), requiring 140 to 400 participants for model structure [22].

### Work Package 1a (WP1a): Moderation

In KAPS, TAPS and STEMS we examined the association between HL (using a validated measure of HL, the Single Item Literacy Screener) [26] and MSK pain and function outcome at follow-up. The HL question in Morris et al. is: “How often do you need to have someone help you when you read instructions on pamphlets, or other written material from your doctor or pharmacy?” [26]. For simplification and clinical interpretation, a cut-off for having ‘limited’ HL was defined as having difficulty or requiring someone to help at least some of the time.

The three datasets included valid scale measurement of pain and function outcomes (0–10 numerical rating scale (in KAPS and TAPS, [27]) and SF-12 (in STEMS, [28]), and have multiple data collection time points to assess the temporal relationships between variables. The final follow-up time point in each study is used to define the outcome time reference in this study: hence, 6 months for KAPS and TAPS and 12 months for STEMS.

Potential confounders accounted for in the analyses were: age; gender; education; employment status; socio-economic status; co-morbidities; living alone; accommodation ownership. Association between HL and pain and function were examined before and after covariate adjustment.

#### Potential moderation

The relationship between HL and the outcomes of pain intensity or physical function may be different within different population socio-demographic subgroups. Hence, we examined whether the same socio-demographic variables (given above) moderated any association between HL and pain / function outcome, which may further help in informing the strategy for targeting of any possible intervention.

#### Statistical analysis

Beyond crude (unadjusted) association, the association of HL with pain intensity and function at final follow-up was analysed using a stepped general linear model, with step 1 adjusting for socio-demographic factors (age, gender, education (age left school, further education, qualifications as an adult)) and socio-economic status, step 2 additionally adjusting for baseline co-morbidities and mental health status, and step 3 further adjusting for baseline pain duration and corresponding baseline values of the outcome measures (pain and function), to estimate the level of independent association of pain and functional outcome with HL.

To examine moderation we modelled the individual interaction effects between each of the baseline socio-demographic / health variables and the HL variable (through inclusion of the interaction term as well as the main effects terms in the regression model e.g. age x literacy included alongside age and literacy terms). Since moderator evaluations are generally under-powered relative to main-effects evaluations we report the interactions that give p-values less than 0.1.

### Work Package 1b (WP1b): Mediation

We conducted a mediation analysis [29, 30] to identify potential intervention targets that may explain the predictive relationship between limited HL and poor outcomes. Several factors from the published literature were considered as potential intervention targets, including pain self-efficacy [31], psychological factors (illness perceptions [32]); pain catastrophizing [33], consultation experience (communication) [34], and sleep problems [35].

Further, we conducted moderation analysis to explore factors that affect the strength of the mediation relationships between HL at baseline, the mediators, and outcomes (pain intensity, physical function). This analysis (moderated mediation) potentially identifies sub-groups of individuals where mediation affect is increased/decreased, which will help support intervention development.

#### Statistical analysis

Structural equation modelling (SEM) was used to conduct the mediation analyses. Unstandardised and standardised model coefficients are presented for the indirect effects through mediation and the affiliated p-values. The percent indirect effect through the mediation variables (relative to the total effect of HL on pain / function outcomes) are presented. The mediation models included age, gender, comorbidity and education/ socio-economic status as covariates.

For those mediator variables accounting for at least a quarter (≥ 25%) of the total effect, further evaluation was carried out to assess whether socio-demographic subgroups based on age, gender, education, co-morbidity and socio-economic status, modified the mediation effect.

All statistical analyses were carried out using IBM SPSS version24, AMOS and STATA_v15.

### Work Package 2 (WP2): Evidence synthesis to identify components of supported self-management suitable for people with limited HL

The objective of WP2 was to summarize evidence from previous trials to identify which types of interventions, or components of interventions, aiming to support self-management of a long-term condition (e.g. diabetes, asthma/COPD, heart failure, inflammatory bowel disease, chronic pain, long-term depression) have been developed specifically for people with limited health literacy and demonstrated to be effective. For example, supported self-management interventions may include components focusing on lifestyle (e.g. improving physical activity); pain education, medication management, or mind–body therapy.

An evidence synthesis was conducted in accordance with PRISMA (Preferred Reporting Items for Systematic Reviews and meta-Analyses) and registered with the International prospective register of systematic reviews (PROSPERO ID: CRD: 42019136424). An experienced information specialist (NC) designed and conducted searches from 2009 until March 2019 in MEDLINE (OVID), EMBASE (OVID), CINAHLPlus (EBSCO), AMED (OVID), PsycINFO (EBSCO) and the Cochrane Library (see Additional file 2).

Given the broad topics of searches we took an efficient approach to identifying relevant RCTs, by conducting (a) searches to identify recent systematic reviews of relevant RCTs (past 10 years), and (b) updated searches to identify more recently published relevant individual RCTs. Additional methods for identifying eligible studies included: (i) checking reference lists of included RCTs and systematic reviews; (ii) consulting experts for additional studies; and (iii) citation tracking of key documents/reports or systematic reviews. We included RCTs of any design (e.g. individual or cluster randomisation, step-wedge design). All other study designs were excluded (e.g. non-randomised controlled studies; cross-sectional designs; case reports).

We included studies of supported self-management interventions developed for adults diagnosed with at least one long-term condition (LTC) (e.g. diabetes, asthma/COPD, heart failure, inflammatory bowel disease, chronic pain, long-term depression) and who had been identified with limited HL using a validated measure. Interventions could include components focusing on lifestyle (e.g. improving physical activity), pain education, medication management, or mind–body therapy. The review included studies conducted in primary care, community care, occupational healthcare or rehabilitation settings, studies conducted in hospital settings were excluded, except for those concerning supported self-management interventions provided at discharge.

Eligibility screening of titles and abstracts, then full texts, was conducted by sets of two review authors independently. Disagreements were resolved by discussion or by consulting a third reviewer where necessary. A standardised data extraction form was developed, piloted and used to collect data for analysis. Study-level data were collected on healthcare setting; selection criteria; population characteristics; characteristics of investigated interventions; process measures (acceptability, feasibility, impact on services); outcome measures; and effect estimates. The TIDIER (Template for Intervention Description and Replication) checklist was used to extract details regarding included interventions [36]. Data extraction and assessment of risk of bias, using the Cochrane risk of bias tool, was undertaken by one review author and checked by a second reviewer. Using the findings of the review we constructed a list of intervention components and modes of delivery that were included in supported self-management interventions shown to be effective (superior) compared to control interventions.

To ensure we had a comprehensive approach to identifying relevant self-management approaches that are relevant and suitable for people with MSK conditions, we also conducted an umbrella review (overview of systematic reviews, PROSPERO: CRD42019136462) which summarised evidence of interventions to support self-management specifically in people with MSK pain, and included a focus on varying levels of health literacy [37]. This work fed into work package 4.

### Work Package 3 (WP3): Qualitative investigation of the experiences of people living with MSK pain, HCPs and experts around self-management of MSK pain

The objective of WP3 was to examine the experiences of people living with MSK pain, HCPs and experts around self-management of MSK pain, and their views on the most appropriate intervention components that are also suitable for people with varying levels of HL.

This work package explored:

- 1. Personal and/or professional experiences around MSK condition self-management.
- 2. Potential targets for intervention for supported self-management and for intervention design identified in WP1.
- 3. Appropriate intervention components and how best to deliver them in primary care for patients with varying levels of HL.

A qualitative design was employed comprising 4 focus groups [38]. Focus group methodology was chosen as it provides a means of eliciting diversity, dimensions and nuances to a problem that one individual may not have thought of, and may result in a very different understanding emerging than had been originally envisaged. The intention of the focus groups was not to generate consensus, but to allow participants to share their own experiences, generating further questions and concepts for discussion [39, 40], and thus generate a richer understanding of the phenomenon.

In addition to a topic guide, we used a case vignette focused on a fictional character ‘Robert’ who has MSK pain and may have problems with their HL. The case vignette was introduced as an ‘ice-breaker’ at the start of each focus group to help facilitate and focus the discussion around what can be a sensitive issue for some participants (Additional file 3). The case vignette and topic guides were refined through advice from our Patient and Public Involvement and Engagement (PPIE) group.

All discussions were audio-recorded with consent, fully transcribed and anonymised before analysis. Researchers also kept detailed field notes which were incorporated into the data analysis. PPIE members were also involved in the analysis process and agreed to the main themes developed.

#### Recruitment

Purposive sampling was used to identify informant rich cases to ensure a wide range of perspectives was included [41].

#### Community members with an MSK condition

We conducted 3 focus groups with two researchers facilitating each group. The focus groups were held at venues in local communities.

Participants were recruited through relevant existing community groups and classes/groups within or recommended by the Workers Education Association (WEA), who have expertise in working in community settings with those with limited HL (see Additional file 4).

#### Professionals

To ensure a broad range of perspectives in our professional stakeholder group, we included a range of HCPs (e.g. GPs, nurses, physiotherapists, rheumatologists, psychologists), experts in HL, and 3rd sector experts working with people with limited HL. Participants were recruited via professional network (see Additional file 4).

#### Eligibility criteria

• Community members with self-reported diagnosis of an MSK condition *or* Professionals whose services engage with those with MSK pain and/or varying levels of HL
• Aged ≥ 18 years
• English speaking
• Able to provide informed consent

#### Data analysis

Reflecting the mixed methods approach to data collection, metaphor elicitation was adopted within a framework approach [42] to thematically analyse the data, emphasising data integration throughout the process of collection and analysis [43]. An iterative approach was undertaken, with analysis beginning from the first data collection and emerging findings checked out in subsequent data collection, and across the two groups. The analysis was carried out by a team of coders to ensure inter-coder reliability [42].

### Work Package 4 (WP4): Triangulation of findings from work packages 2 and 3 and modified Delphi consensus study to produce an evidence-based logic model

The objective of WP4 was to triangulate findings from WP2 and WP3 and determine a consensus from patients, HCPs and experts on the factors that should be included in better ways of supporting people to manage their MSK condition that is also suitable for people with varying levels of limited HL.

### Work package 4a (WP4a): Triangulation of findings and a modified online Delphi consensus study

Firstly, an evidence table was produced through triangulation of the findings from work packages 2 and 3 by the research team. ‘Triangulation’ describes the process of studying a problem using different methods to gain a more complete picture [44]. Findings were grouped together before being explored for commonalities and divergence. They were then triangulated into a final evidence table.

The evidence table was then used to create a number of statements which were then the focus of an online modified Delphi consensus study, where HCPs and experts from the third sector were asked to rank the importance of the statements as potential components and modes of delivery for an intervention to provide supported self-management for those with MSK pain and varying levels of HL.

The Delphi technique is considered a reliable approach to developing new concepts and setting the direction of future research [45], and has been commonly used to establish consensus across a variety of disciplines in the field of MSK health [46, 47].

#### Recruitment

Invitations to participate, including the participant information leaflet and an online link to participate, were sent via email to the professional networks of the research team which included HCPs and third sector experts.

#### Data collection

The modified Delphi used the online Keele Health Survey (LimeSurvey) hosted by Keele University. Participants were asked to rank the importance of the evidence statements produced from the triangulation of the findings from work packages 2 and 3. The importance of the statements were ranked on a 4-point Likert scale (i.e. definitely include, probably include, probably not include, definitely not include) (see Additional file 5).

#### Data analysis

A consensus level of 80% agreement for inclusion (definitely include) for each evidence statement across all respondents after each round was decided upon a priori, following a review of the literature [47, 48]. The statements that didn’t reach consensus in round 1 were re-ranked in round 2.

Following the Delphi, we took the evidence statements with 80% consensus for inclusion and mapped them to the findings of the systematic review and assessed the overall confidence in the evidence by grading them as high, moderate, low or very low using an approach modified from GRADE (Grading of Recommendations Assessment, Development, and Evaluation) methodology [49].

### Work package 4b (WP4b): Production of the logic model

The research team used the agreed components identified through the Delphi to produce a logic model that explains how potential intervention components could address potential mediators, leading to improved health outcomes for people who have MSK pain and varying levels of HL.

### Patient and public involvement and engagement (PPIE)

Involvement was embedded in all stages of the EASIER study. The study concept and design was co-produced with our PPIE members. PPIE members advised on the participant facing documents, the topic guides and the case vignettes for WP3. They were also asked to reflect on the proposed recruitment strategy and location of the focus groups. PPIE input was obtained at an analysis workshop focused on the WP3 data. A final workshop with PPIE members took place to allow them to share their perspectives on the components of interventions that were incorporated into the logic model based on the modified online Delphi.

## Results

### Work Package 1 (WP1): Analysis of the relationship between HL and MSK health outcomes (objective 1)

#### Work Package 1a (WP1a): Moderation

Table 1 provides data on the associations between HL and socio-demographic and baseline and follow up health status variables across the three datasets. Compared to people with adequate HL, people with limited HL had a higher average age, lesser educational background and socio-economic profile, and receive less help and support with emotional needs and daily tasks. People with limited HL had significantly worse health status at both baseline and follow up: worse pain scores, worse function, worse mental health and higher kinesiophobia (fear of pain due to movement) and were more likely to have had their current pain for > 3 months.

**Table 1:** Characteristics of study population by baseline HL response categories

	**Need help reading health-related materials**							
	**Never**	**Rarely**	**Sometimes**	**Often**	**Always**	**Adequate HL** ^**b**^	**Inadequate HL** ^**c**^	**Total Score**
**KAPS**								
Overall, n (%)	1321 (70.3)	234 (12.4)	185 (9.8)	72 (3.8)	68 (3.6)	1555 (82.7)	325 (17.3)	1880^a^
Age (years), mean (SD)^1^	57.4 (15.6)	60.5 (16.7)	61.2 (17.5)	59.1 (16.2)	58.5 (17.7)	57.9 (15.8)	60.2 (17.2)	58.3 (16.1)
Females, n (%)	812 (61.5)	133 (56.8)	106 (57.3)	50 (69.4)	37 (54.4)	945 (60.8)	193 (59.4)	1138 (60.5)
Education—Age left school (≤ 15 years), n (%) ^3^	432 (33.2)	113 (49.1)	94 (52.8)	28 (40.6)	42 (65.6)	545 (35.6)	164 (52.7)	709 (38.4)
Full-time education, n (%)	454 (34.6)	44 (19.0)	31 (16.9)	15 (21.1)	7 (10.4)	498 (32.3)	53 (16.5)	551 (29.6)
Gained qualifications as adult, n (%) ^3^	782 (61.3)	95 (43.0)	63 (36.2)	22 (32.4)	21 (32.3)	877 (58.6)	106 (34.5)	983 (54.5)
Currently employed, n (%) ^3^	616 (48.4)	66 (29.1)	42 (23.2)	13 (19.1)	6 (9.5)	682 (45.4)	61 (19.6)	743 (41.0)
Living alone, n (%) ^3^	243 (18.5)	58 (24.9)	53 (28.8)	17 (23.9)	22 (32.4)	301 (19.5)	92 (28.5)	393 (21.0)
No emotional support, n (%) ^3^	90 (6.9)	19 (8.2)	22 (12.1)	10 (14.3)	6 (9.0)	109 (7.1)	38 (11.9)	147 (7.9)
No daily tasks support, n (%) ^3^	121 (9.2)	22 (9.4)	19 (10.3)	9 (12.5)	5 (7.4)	143 (9.3)	33 (10.2)	176 (9.4)
≥ 2 co-morbidities, n (%) ^3^	334 (25.3)	86 (36.8)	82 (44.3)	26 (36.1)	40 (58.8)	420 (27.0)	148 (45.5)	568 (30.2)
Pain duration > 3 months, n (%)^1^	853 (65.7)	160 (70.2)	129 (73.3)	53 (74.7)	51 (77.3)	1013 (66.4)	233 (74.4)	1246 (67.8)
Mental health (SF12-MCS), mean (SD)								
Baseline^3^	67.8 (21.0)	59.0 (20.8)	52.3 (21.7)	47.6 (23.9)	40.1 (25.0)	66.5 (21.2)	48.7 (23.3)	63.4 (22.6)
6 months^3^	73.4 (19.2)	67.2 (21.5)	59.1 (22.7)	55.0 (25.7)	46.7 (28.4)	72.5 (19.6)	56.8 (24.2)	70.3 (21.0)
Pain (NRS 0–10 scale), mean (SD)								
Baseline^3^	5.0 (2.3)	5.6 (2.2)	6.5 (2.1)	6.2 (2.3)	7.0 (2.2)	5.1 (2.3)	6.6 (2.2)	5.3 (2.4)
6 months^3^	3.9 (3.0)	4.7 (2.9)	5.7 (2.9)	5.9 (2.7)	6.7 (2.5)	3.9 (2.7)	5.8 (2.6)	4.1 (2.8)
Function (SF12-PCS), mean (SD)								
Baseline^3^	37.4 (10.1)	35.3 (9.7)	31.6 (9.1)	32.3 (8.6)	31.1 (10.4)	37.1 (10.1)	31.7 (9.2)	36.2 (10.2)
6 months^3^	40.1 (11.4)	37.1 (10.5)	31.9 (9.2)	32.3 (9.3)	31.8 (10.0)	39.7 (11.3)	32.0 (9.3)	38.6 (11.4)
**TAPS**								
Overall, n (%)	410 (79.5)	62 (12.0)	28 (5.4)	8 (1.6)	8 (1.6)	472 (91.5)	44 (8.5)	516^a^
Age (years), mean (SD) ^2^	60.4 (14.5)	61.9 (16.0)	65.3(14.8)	66.9 (11.1)	74.5 (17.4)	60.6 (14.7)	67.3 (14.8)	61.2 (14.8)
Females, n (%)	253 (61.7)	33 (53.2)	19 (67.9)	7 (87.5)	3 (37.5)	286 (60.6)	29 (65.9)	315 (61.0)
IMD rank, mean (SD)	23,087 (6459)	23,179 (6325)	20,420 (7717)	22,193 (9848)	24,203 (6809)	23,099 (6435)	21,430 (7933)	22,956 (6584)
Social class (SOC-2010), n (%)^1^								
Class 1–2	136 (34.6)	16 (26.2)	3 (13.0)	1 (14.3)	2 (28.6)	152 (33.5)	6 (16.2)	158 (32.2)
Class 3–4	123 (31.3)	12 (19.7)	8 (34.8)	3 (42.9)	0 (0.0)	135 (29.7)	11 (29.7)	146 (29.7)
Class 5–9	134 (34.1)	33 (54.1)	12 (52.2)	3 (42.9)	5 (71.4)	167 (36.8)	20 (54.1)	187 (38.1)
Currently employed, n (%)^2^	192 (48.1)	29 (47.5)	6 (22.2)	3 (37.5)	1 (12.5)	221 (48.0)	10 (23.3)	231 (45.9)
Living alone, n (%)^1^	62 (15.1)	10 (16.1)	6 (21.4)	4 (50.0)	2 (25.0)	72 (15.3)	12 (27.3)	84 (16.3)
GP provided written information, n (%)	162 (40.9)	31 (52.5)	11 (42.3)	3 (42.9)	1 (16.7)	193 (42.4)	15 (38.5)	208 (42.1)
HP-Behaviour index, mean (SD)	47.6 (16.0)	50.6 (14.8)	47.1 (17.1)	49.9 (23.4)	46.5 (17.8)	48.0 (15.9)	47.5 (18.1)	47.9 (16.0)
≥ 2 co-morbidities, n (%) ^3^	120 (29.3)	27 (43.5)	16 (57.1)	4 (50.0)	7 (87.5)	147 (31.1)	27 (61.4)	174 (33.7)
Pain duration > 3 months, n (%)	40 (58.5)	38 (61.3)	20 (71.4)	5 (62.5)	5 (62.5)	278 (58.9)	30 (68.2)	308 (59.7)
Mental health (TSK), mean (SD)								
Baseline TSK^3^	24.0 (6.7)	25.6 (5.7)	27.5 (8.7)	29.4 (6.8)	28.2 (6.7)	24.2 (6.6)	27.0 (7.9)	24.5 (6.8)
6 months TSK^3^	22.6 (7.2)	24.3 (4.9)	26.9 (8.2)	31.5 (8.4)	27.7 (8.5)	22.8 (6.9)	27.9 (8.2)	23.2 (7.2)
Pain (NRS 0–10 scale), mean (SD)								
Baseline^3^	6.0 (2.3)	6.5 (1.9)	7.5 (2.0)	6.4 (3.3)	7.5 (1.8)	6.1 (2.2)	7.3 (2.2)	6.2 (2.3)
6 months^3^	3.8 (2.9)	4.7 (2.7)	5.9 (2.9)	7.4 (2.5)	5.0 (1.7)	3.9 (2.9)	6.1 (2.7)	4.1 (2.9)
Physical function (MSK-HQ), mean (SD)								
Baseline^3^	30.7 (10.1)	27.3 (9.5)	22.2 (10.8)	22.1 (15.8)	20.7 (8.5)	30.2 (10.1)	21.9 (11.4)	29.6 (10.4)
6 months^3^	39.2 (11.2)	33.7 (12.7)	26.0 (12.7)	26.1 (17.7)	27.8 (11.9)	38.5 (11.5)	26.2 (13.3)	37.4 (12.2)
**STEMS**								
Overall, n (%)	418 (43.0)	207 (21.3)	265 (27.3)	64 (6.6)	17 (1.8)	625 (64.4)	346 (35.6)	971^a^
Age (years), mean (SD) ^3^	55.9 (14.5)	56.2 (15.2)	59.2 (14.4)	60.1 (17.7)	60.7 (13.6)	56.0 (14.8)	59.5 (15.0)	57.2 (14.9)
Females, n (%)	240 (57.4)	122 (58.9)	146 (55.1)	38 (59.4)	8 (47.1)	362 (57.9)	192 (55.5)	554 (57.1)
Non-white ethnic, n (%)	3 (0.7)	3 (1.5)	1 (0.4)	0 (0.0)	1 (5.9)	6 (1.0)	2 (0.6)	8 (0.8)
IMD rank, mean (SD) ^3^	14,662 (7017)	13,996 (7396)	12,829 (7141)	12,265 (7106)	10,030 (6272)	14,441 (7145)	12,587 (7103)	13,781 (7182)
Education – No academic/work qualification, n (%) ^3^	63 (16.9)	36 (20.9)	101 (43.7)	27 (50.0)	9 (60.0)	99 (18.2)	137 (45.7)	236 (27.9)
Accommodation not owned, n (%) ^3^	85 (20.9)	53 (26.2)	89 (34.4)	31 (50.8)	8 (53.3)	138 (22.7)	128 (38.2)	266 (28.2)
Currently employed, n (%) ^3^	214 (51.4)	101 (49.3)	91 (34.6)	21 (35.6)	3 (17.6)	315 (50.7)	115 (33.9)	430 (44.8)
≥ 2 co-morbidities, n (%) ^3^	224 (53.6)	118 (57.0)	168 (63.4)	46 (71.9)	15 (88.2)	342 (54.7)	229 (66.2)	571 (58.8)
Pain duration > 3 months, n (%)^1^	248 (62.0)	116 (58.6)	159 (64.9)	47 (77.0)	14 (82.4)	364 (60.8)	220 (68.1)	584 (63.4)
Mental health (SF-12 MCS), mean (SD)								
Baseline^3^	47.0 (13.5)	43.4 (12.6)	40.7 (13.7)	36.8 (13.9)	35.4 (13.9)	45.8 (13.3)	39.7 (13.9)	43.7 (13.8)
12 months^3^	48.4 (12.2)	46.2 (12.3)	42.2 (13.8)	35.0 (15.0)	34.0 (15.8)	47.7 (12.2)	40.7 (14.4)	45.6 (13.3)
Pain (SF-12 BP), mean (SD)								
Baseline^3^	44.4 (24.0)	42.0 (22.7)	37.2 (22.2)	33.5 (25.0)	22.0 (14.1)	43.6 (23.6)	35.7 (22.6)	40.8 (23.5)
12 months^3^	58.3 (27.3)	55.5 (27.7)	45.9 (25.1)	36.7 (21.5)	22.5 (18.2)	57.4 (27.4)	43.1 (24.8)	53.0 (27.5)
Physical functioning (SF-12 PCS), mean (SD)								
Baseline^3^	37.7 (10.1)	36.7 (10.2)	34.5 (9.4)	32.8 (9.3)	27.0 (8.7)	37.3 (10.1)	33.8 (9.5)	36.1 (10.0)
12 months^3^	41.1 (11.8)	40.2 (11.6)	36.3 (10.8)	32.1 (7.7)	27.1 (6.0)	40.9 (11.7)	35.2 (10.4)	39.1 (11.6)

Social Class (SOC-2010): 1 Managers, directors and senior officials; 2 Professional occupations; 3 Associate professional and technical occupations; 4 Administrative and secretarial occupations; 5 Skilled trades occupations; 6 Caring, leisure and other service occupations; 7 Sales and customer service occupations; 8 Process, plant and machine operatives; 9 Elementary occupations

*SF12 *Short-Form_12 (MCS = Mental Component Scale, *PCS *Physical Component Scale; 0 = worst health status, 100 = best health status); *NRS *Numerical Rating Scale (0–10 Pain scale; 0 = no pain, 10 = worst pain); *HP *Behaviour Index = Health Professional Behaviour & Communication index: 0 = Poor behaviour/communication, 72 = Excellent behaviour/communication; *TSK *Tampa Scale of Kinesiophobia (17–68 scale; 0 = no fear, 68 = most fear); *MSK-HQ *MuSKuloskeletal Health Questionnaire)

^1^*p* < 0.05, ^2^*p* < 0.01, ^3^*p* < 0.001 by one-way ANOVA trend test with linear contrast (1df) or chi square test / chi square test for trend for trend for comparison across the five subcategories of health literacy

^a^Missing data for the health literacy question (*n* = 10 KAPS; *n* = 8 TAPS; *n* = 7 STEMS)

^b^Adequate HL = never, rarely need help

^c^Inadequate HL = sometimes, often, always need help

Table 2 presents the unadjusted mean difference estimates which show that people with limited HL had significantly worse health status than those with adequate HL. The differences were largely retained despite adjustment for socio-demographic variables. Further adjustment for co-morbidity and baseline mental health status reduced the differences to about half the size of the unadjusted difference in many of the analyses, and some differences persisted after further adjustment for baseline score and pain duration.

**Table 2:** Differences in physical function and pain intensity scores between patients with inadequate and adequate HL

	**Unadjusted**		Adjusted*		Adjusted**	
	**Mean difference (95% CI)**	**SMD (95% CI)**	**Mean difference (95% CI)**	**SMD (95% CI)**	**Mean difference (95% CI)**	**SMD** **(95% CI)**
**KAPS**						
** Function [SF12-PCS]**						
Baseline	-5.4 (-6.7, -4.2)^3^	-0.53 (-0.66, -0.41)	-4.4 (-5.8, -3.1)^3^	-0.43 (-0.56, -0.29)	-2.9 (-4.3, -1.6)^3^	-0.27 (-0.41, -0.15)
6 months	-7.7 (-9.5, -5.8)^3^	-0.75 (-0.93, -0.57)	-5.7 (-7.6, -3.9)^3^	-0.56 (-0.75, -0.37)	-2.7 (-4.6, -0.8)^2^	-0.26 (-0.45, 0.08)
** Pain (0–10 NRS-scale)**						
Baseline	1.5 (1.2, 1.8)^3^	0.62 (0.46, 0.74)	1.2 (0.9, 1.4)^3^	0.48 (0.36, 0.60)	0.5 (0.2, 0.8)^2^	0.20 (0.09, 0.33)
6 months	2.0 (1.5, 2.4)^3^	0.82 (0.64, 1.00)	1.6 (1.2, 2.1)^3^	0.67 (0.49, 0.86)	0.7 (0.3, 1.2)^2^	0.30 (0.12, 0.49)
**TAPS**						
** Function [MSK-HQ]**						
Baseline	-8.3 (-11.7, -5.0)^3^	-0.80 (-1.13, -0.48)	-7.2 (-10.9, -3.5)^3^	-0.69 (-1.05, -0.34)	-3.6 (-6.9, -0.2)^1^	-0.34 (-0.66, -0.02)
6 months	-12.2 (-16.2, -8.3)^3^	-1.17 (-1.56, -0.80)	-10.9 (-15.3, -6.4)^3^	-1.04 (-1.46, -0.61)	-8.2 (-12.4, -4.1)^3^	-0.70 (-1.10, -0.31)
** Pain [0–10 NRS-scale]**						
Baseline	1.2 (0.5, 1.9)^3^	0.52 (0.22, 0.82)	1.0 (0.3, 1.8)^2^	0.44 (0.11, 0.78)	0.6 (-0.2, 1.4)	0.27 (-0.08, 0.61)
6 m (Post)	2.2 (1.3, 3.2)^3^	0.96 (0.55, 1.37)	1.8 (0.8, 2.9)^3^	0.77 (0.32, 1.23)	1.5 (0.5, 2.6)^2^	0.43 (0.00, 0.87)
**STEMS**						
** Function [SF12-PCS]**						
Baseline	-3.5 (-4.8, -2.2)^3^	-0.35 (-0.48, -0.22)	-2.4 (-3.9, -1.0)^2^	-0.25 (-0.39, -0.11)	-1.7 (-3.1, -0.3)^1^	-0.17 (-0.31, -0.03)
12 months	-5.7 (-7.6, -3.7)^3^	-0.57 (-0.76, -0.37)	-3.6 (-5.6, -1.5)^2^	-0.36 (-0.56, -0.15)	-2.3 (-4.3, -0.3)^1^	-0.23 (-0.43, -0.03)
** Pain [SF12-BP]**						
Baseline	-7.9 (-10.9, -4.8)^3^	-0.34 (-0.46, -0.20)	-5.0 (-8.5, -1.5)^2^	-0.22 (-0.36, -0.07)	-1.1 (-4.3, 2.0)	-0.05 (-0.18, 0.08)
12 months	-14.3 (-18.7, -9.9)^3^	-0.61 (-0.80, -0.42)	-10.7 (-15.5, -5.9)^3^	-0.46 (-0.66, -0.26)	-6.9 (-11.4, -2.3)^2^	-0.29 (-0.48, -0.10)

Inadequate HL = often, always, sometimes need help; Adequate HL = rarely, never need help. Mean difference from linear regression analyses calculated as: mean score (inadequate HL group) minus mean score (adequate HL (reference group)). SMD = Standardised mean difference (mean difference / baseline SD)

[KAPS] * Adjusted for age, gender, age left school, further education, qualifications as adult, currently employed, living alone status; ** additionally adjusted for baseline co-morbidities and mental health score. Further adjustment for pain duration and baseline score: PCS at 6 months, mean difference = 0.7 (95%CI -0.6, 2.0) (*P* = 0.30); Pain at 6 months, mean difference = 0.3 (-0.1, 0.6) (*P* = 0.18)

[TAPS] * Adjusted for age, gender, IMD rank, Social class category, currently employed, living alone status; ** additionally adjusted for co-morbidities and mental health (TSK score). Further adjustment for pain duration and baseline score: MSK-HQ at 6 months, mean difference = -5.9 (-9.9, -1.8) (*P* = 0.005); Pain at 6 months, mean difference = 1.3 (0.3, 2.2) (*P* = 0.012)

[STEMS] * Adjusted for age, gender, IMD rank, education, accommodation owned, currently employed; ** additionally adjusted for baseline co-morbidities and mental health score. Further adjustment for pain duration and baseline score: PCS at 12 months, mean difference = -1.4 (-3.0, 0.2) (*P* = 0.093); Pain at 12 months, mean difference = -6.4 (-10.6, -2.1) (*P* = 0.003)

SF12 = Short-Form_12 (MCS = Mental Component Scale, PCS = Physical Component Scale; 0 = worst health status, 100 = best health status); NRS = Numerical Rating Scale (0–10 Pain scale; 0 = no pain, 10 = worst pain); HP-Behaviour Index = Health Professional Behaviour & Communication index (0–72 scale: 0 = Poor behaviour/communication, 72 = Excellent behaviour/communication); TSK = Tampa Scale of Kinesiophobia (17–68 scale; 0 = no fear, 68 = most fear); MSK-HQ = MuSKuloskeletal Health Questionnaire)

CI = Confidence Interval. ^1^ *p* < 0.05, ^2^ *p* < 0.01, ^3^ *p* < 0.001 by linear regression

The moderation analysis (see Additional file 6) demonstrated there were few clear and consistent indicators of a moderation effect of limited HL by increased age; lower-level of education; deprivation indices; non-employment; lower baseline physical and mental health status; longer duration of symptoms, or comorbidity. Within the KAPS dataset only, there was a statistically significant interaction effect for HL with school-leaving and full-time education variables, where the signs for the coefficients were opposite to those for the individual variables (pointing towards a possible lowering impact of combined limited HL and lower-level education than for the sum of the individual effects).

#### Work Package 1b (WP1b): Mediation

Psychological factors were significant mediators: indicating high indirect effects for the relationship between HL and pain / function outcomes, which were mediated through these factors; to the extent of 47–100% indirect effects within KAPS, 33–88% within TAPS, and 79–95% within STEMS (see Additional file 6). Mediation through limited HL influence on increased catastrophizing (leading to poorer pain / function outcomes at follow up) was significantly elevated across the following subgroups in KAPS: age < 65 years, males, co-morbidity, full-time education, mild pain at baseline. Similar moderation of the mediation with heightened difference for subgroups aged < 65 years and males were also observed for pain self-efficacy and illness-perceptions mediators within KAPS. There was little evidence of moderation of mediation within TAPS and STEMS (see Additional file 7).

### Work Package 2 (WP2): Evidence synthesis to identify components of supported self-management suitable for people with limited HL (objective 2)

**Fig. 2:**
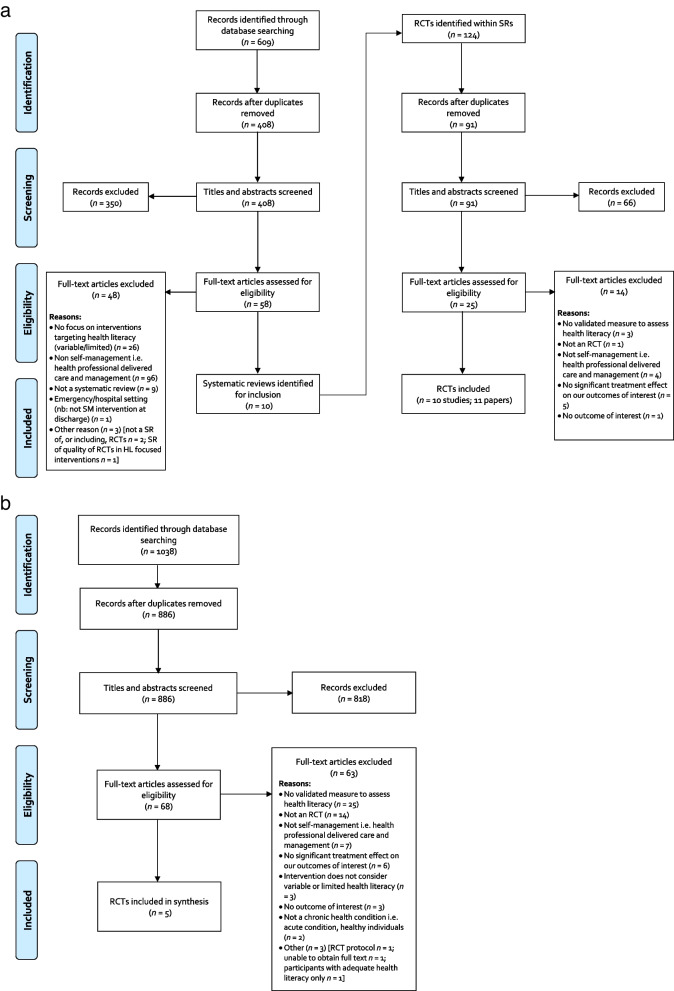
**a** PRISMA Flow Diagram for Systematic Reviews. **b** PRISMA Flow Diagram for Randomised Controlled Trials

The search to identify recent systematic reviews of relevant RCTs (past 10 years) resulted in the identification of 408 unique citations. Screening and full text eligibility assessment removed 398 systematic reviews, leaving 10 for inclusion. From these 10 systematic reviews, 91 unique citations were identified and 10 RCTs (11 papers) were deemed eligible (see Fig. 2a).

The search to identify more recently published relevant individual RCTs resulted in the identification of 886 unique citations. Screening and full text eligibility assessment removed 881 RCTs, leaving 5 for inclusion (see Fig. 2b).

The characteristics of the studies included are described in Table 3. The majority of the studies were from the United States and focused on a range of LTCs such as diabetes, hypertension and heart failure. An overview of the components of supported self-management interventions for people with LTCs and varying levels of HL that demonstrated significant effects are described in Table 4. These components focused on mode of delivery, type of HCP delivering the intervention, training for intervention deliverers, intervention materials and patient knowledge, understanding and support. The TIDIER checklist was completed to support the completeness in the reporting of the interventions identified (See Additional file 8).

**Table 3:** Characteristics of the 15 studies (16 papers included)

Characteristic	*n* studies (articles)	Reference
**Country**		
USA	12 (13)	Bosworth, 2018; 2009; 2005 [50–52]; Carroll, 2019 [53]; Cavanaugh, 2009 [54]; DeWalt, 2012; 2006 [55, 56]; Kalichman, 2013 [57]; Kiser, 2012 [58]; Murray, 2007 [59]; Press, 2016 [60]; Schillinger, 2008; 2009 [61, 62]
Iran	1	Negarandeh, 2013 [63]
The Netherlands	1	Eikelenboom, 2016 [64]
South Korea	1	Lee, 2017 [65]
**Condition**		
Diabetes (type II)	3 (4)	Lee, 2017 [65]; Negarandeh, 2013 [63]; Schillinger, 2008;2009 [61, 62]
Heart failure	3	DeWalt, 2012; 2006 [55, 56]; Murray, 2007 [59]
Hypertension	3	Bosworth, 2018; 2009; 2005 [50–52]
HIV	2	Carroll, 2019 [53]; Kalichman, 2014 [57]
Asthma or COPD	1	Press, 2016 [60]
COPD; chronic bronchitis, emphysema	1	Kiser, 2012 [58]
Diabetes (type I & II)	1	Cavanaugh, 2009 [54]
Chronic disease (Diabetes, CVD, COPD, Asthma)	1	Eikelenboom, 2016 [64]
**Health literacy tool**		
sTOFHLA	7 (8)	DeWalt, 2012; 2006 [55, 56]; Eikelenboom, 2016 [64]; Kiser, 2012 [58]; Murray, 2007 [59]; Press, 2016 [60]; Schillinger, 2008; 2009 [61, 62]
REALM	4	Bosworth, 2018; 2009; 2005 [50–52]; Cavanaugh, 2009 [54]
TOFHLA	2	Kalichman, 2014 [57]; Negarandeh, 2013 [63]
eHEALS	1	Carroll, 2019 [53]
Korean Health Literacy Assessment tool	1	Lee, 2017 [65]
**Mean age**		
40–49 years	2	Kalichman, 2014[57]; Press, 2016 (median) [60]
50–59 years	4 (5)	Carroll, 2019 [53]; Cavanaugh, 2009[54]; Negarandeh, 2013 [63]; Schillinger, 2008; 2009 [61, 62]
60–69 years	8	Bosworth, 2018; 2009; 2005 [50–52]; DeWalt, 2012; 2006 [55, 56]; Eikelenboom, 2016 [64]; Kiser, 2012 [58]; Murray, 2007 [59]
70–79 years	1	Lee, 2017 [65]
**% Female**		
0–19%	2	Bosworth, 2018; 2005 [50, 52]
20–39%	2	Kalichman, 2014 [57]; Carroll, 2019 [53]
40–59%	6 (7)	DeWalt, 2012, 2009 [55, 56]; Eikelenboom, 2016 [64]; Lee, 2017[65]; Negarandeh, 2013[63]; Schillinger, 2008, 2009 [61, 62]
60–79%	5	Bosworth, 2009 [51]; Cavanaugh, 2009 [54]; Kiser, 2012 [58]; Murray, 2007 [59]; Press, 2016 [60]
**Study duration**		
0–3 months	4	Kiser, 2012 [58]; Lee, 2017 [65]; Negarandeh, 2013 [63]; Press, 2016 [60]
4–6 months	1	Eikelenboom, 2016 [64]
7–12 months	8 (9)	Bosworth, 2018 [52]; Carroll, 2019 [53]; Cavanaugh, 2009 [54]; DeWalt, 2012; 2006 [55, 56]; Kalichman, 2014 [57]; Murray, 2007 [59]; Schillinger, 2008; 2009 [61, 62]
13–24 months	2	Bosworth, 2009; 2005 [50, 51]
**Outcome type of interest reported**		
Disease specific	15 (16)	Bosworth, 2018; 2009; 2005 [50–52]; Carroll, 2019 [53]; Cavanaugh, 2009 [54]; DeWalt, 2012; 2006 [55, 56]; Eikelenboom, 2016 [64]; Kalichman, 2014 [57]; Kiser, 2012 [58]; Lee, 2017 [65]; Murray, 2007 [59]; Negarandeh, 2013 [63]; Press, 2016 [60]; Schillinger, 2008; 2009 [61, 62]
Self-efficacy	6 (7)	Bosworth, 2005 [50]; Carroll, 2019 [53]; Cavanaugh, 2009 [54]; DeWalt, 2006 [55]; Lee, 2017 [65]; Schillinger, 2008; 2009[61, 62]
Quality of life	5 (6)	Carroll, 2019 [53]; DeWalt, 2012; 200,619,18]; Murray, 2007 [59]; Schillinger, 2008; 2009 [61, 62]
Physical activity	3 (4)	Bosworth, 2009 [50]; Eikelenboom, 2016 [64]; Schillinger, 2008; 2009 [61, 62]
Patient enablement	2	Carroll, 2019 [53]; Eikelenboom, 2016 [64]
**Number of trial arms/interventions**		
2	11	Bosworth, 2018; 2005 [50, 52]; Carroll, 2019 52]; Cavanaugh, 2009 [54]; DeWalt, 2012; 2006 [55, 56]; Eikelenboom, 2016[64]; Kiser, 2012 [58]; Lee, 2017 [65]; Murray, 2007 [59]; Press, 2016 [60]
3	3 (4)	Kalichman, 2014 [57]; Negarandeh, 2013 [63]; Schillinger, 2008; 2009 [61, 62]
4	1	Bosworth, 2009 [51]

**Table 4:** Overview of components of interventions shown to have significant intervention effects. *n* refers to the number of interventions (of a possible 18 identified across 15 studies (16 articles))

Characteristic	*n* of 18 intervention studies	Reference
**Intervention delivery: Modes**		
Face-to-face: individual	14 (12)	Bosworth 2018; 2009 [51, 52]; Carroll 2019 [53]; Cavanaugh 2009[54]; DeWalt 2006 [55]; Eikelenboom 2016 [64]; Kalichman 2014 [57]; Kiser 2012 [38]; Murray 2007 [59]; Negarandeh 2013 [63]; Press 2016 [60]; Schillinger 2008; 2009 [61, 62]
Remote: 1–2-1 phone calls	7 (7)	Bosworth 2018; 2009; 2005 [50–52]; DeWalt 2012; 2006 [55, 56]; Lee 2017 [65]; Schillinger 2008; 2009 [61, 62]
Face-to-face: group	3 (3)	Carroll 2019 [53]; Lee 2017 [65]; Schillinger 2008; 2009 [61, 62]
Remote: automated phone calls	1 (1)	Schillinger 2008; 2009 [61, 62]
Single mode (i.e. 1 of above) only	11 (9)	Bosworth 2005 [50]; Cavanaugh 2009 [54]; DeWalt 2012 [56]; Eikelenboom 2016 [64]; Kalichman 2014 [57]; Kiser 2012 [58]; Murray 2007 [59]; Negarandeh 2013 [63]; Press 2016 [60]
Combination modes (i.e. 2 of above)	7 (6)	Bosworth 2018; 2009 [51, 52]; Carroll 2019 [53]; DeWalt 2006 [55]; Lee 2017 [65]; Schillinger 2008;2009 [61, 62]
Mode type		
F2F (individual and/or group) only	11 (9)	Carroll 2019 [53]; Cavanaugh 2009 [54]; Eikelenboom 2016 [64]; Kalichman 2014 [57]; Kiser 2012 [58]; Murray 2007 [59]; Negarandeh 2013 [63]; Press 2016 [60]; Schillinger 2008; 2009 [61, 62]
Remote (1–2-1 call and/or automated) only	3 (3)	Bosworth 2005 [50]; DeWalt 2012 [55]; Schillinger 2008; 2009 [61, 62]
Combination F2F & remote	4 (4)	Bosworth 2018; 2009 [51, 52]; DeWalt 2006 [55]; Lee 2017 [65]
**Intervention delivery: Health care practitioners**		
Nurse	8 (7)	Bosworth 2009; 2005[51, 52]; Cavanaugh 2009 [54]; Eikelenboom 2016 [64]; Lee 2017 [65]; Negarandeh 2013 [63]; Schillinger 2008; 2009 [61, 62]
Educator (health, peer)	4 (4)	Carroll 2019 [53]; DeWalt 2012; 2006 [55, 56]; Schillinger 2008; 2009 [61, 62]
Pharmacist	5 (5)	Bosworth 2018 [50]; Cavanaugh 2009 [54]; DeWalt 2006 [55]; Murray 2007 [59]; Schillinger 2008; 2009 [61, 62]
Researcher (research assistant, educator)	3 (3)	Bosworth 2009 [51]; Kiser 2012 [58]; Press 2016 [60]
Counsellor	1 (1)	Kalichman 2014 [57]
Dietitian	1 (1)	Cavanaugh 2009 [54]
Primary care physician	1 (1)	Schillinger 2008;2009 [61, 62]
Staff coach	1 (1)	Carroll 2019 [53]
Program coordinator	1 (1)	DeWalt 2006 [55]
Single practitioner type	13 (11)	Bosworth 2018; 2005 [50, 52]; DeWalt 2006 [55]; Eikelenboom 2016 [64]; Kalichman 2014 [57]; Kiser 2012 [58]; Lee 2017 [65]; Murray 2007 [59]; Negarandeh 2013[63]; Press 2016 [60]; Schillinger 2008; 2009 [61, 62]
Multiple practitioner types	5 (5)	Bosworth 2009 [51]; Carroll 2019 [53]; Cavanaugh 2009 [54]; DeWalt 2006 [55]; Schillinger 2008; 2009 [61, 62]
Practitioner group		
Health care professional (nurse, pharmacist, GP/PCP, dietitian)	12 (10)	Bosworth 2018; 2009; 2005 [50–52]; Cavanaugh 2009 [54]; DeWalt 2006 [55]; Eikelenboom 2016 [64]; Lee 2017 [65]; Murray 2007 [59]; Negarandeh 2013 [63]; Schillinger 2008; 2009 [61, 62]
Community advisor (Educator (health, peer), counsellor, program co-ordinator, staff coach)	6 (5)	Carroll 2019 [53]; DeWalt 2012; 2006 [55, 56]; Kalichman 2014 [57]; Schillinger 2008; 2009 [61, 62]
Researcher (research assistant, research educator)	3 (3)	Bosworth 2009 [51]; Kiser 2012 [58]; Press 2016 [60]
No. of practitioner groups involved		
Single practitioner group	15 (13)	Bosworth 2018; 2005 [50, 52]; Carroll 2019 [53]; Cavanaugh 2009 [54]; DeWalt 2012[55]; Eikelenboom 2016 [64]; Kalichman 2014 [57]; Kiser 2012 [58]; Lee 2017 [65]; Murray 2007 [59]; Negarandeh 2013 [63]; Press 2016 [60]; Schillinger 2008; 2009 [61, 62]
Combination of practitioner groups	3 (3)	Bosworth 2009 [51]; DeWalt 2006 [55]; Schillinger 2008; 2009 [61, 62]
**Education/Support/Training mode for intervention deliverers (intervention components)**		
Pre-onset of study/intervention	9 (8)	Carroll 2019 [53]; Cavanaugh 2009 [54]; DeWalt 2012 [56]; Eikelenboom 2016[64]; Kalichman 2014[57]; Kiser 2012 [58]; Murray 2007 [59]; Press 2016 [60]
Ongoing	4 (3)	DeWalt 2012 [56]; Eikelenboom 2016 [64]; Kalichman 2014 [57]
**Intervention materials: Intervention deliverers**		
Manuals, Toolkits, and pre-determined scripts	5 (5)	Bosworth 2005 [50]; Carroll 2019 [53]; Cavanaugh 2009 [54]; Eikelenboom 2016 [64]; Murray 2007 [59]
Patient personality/psychological profile	1 (1)	Eikelenboom 2016 [64]
Computer software (monitoring)	1 (1)	Murray 2007 [59]
**Intervention materials: Patients**		
Handouts, Written instructions, Laminated cards (written)	6 (6)	Bosworth 2005 [50]; Kalichman 2014 [57]; Kiser 2012[58]; Murray 2007 [59]; Negarandeh 2013 [63]; Press 2016 [60]
Manual, Educational book, Workbook,	5 (5)	Bosworth 2018 [52]; Cavanaugh 2009 [54]; DeWalt 2012; 2006 [55, 56]; Lee 2017 [65]
Logbook, Notebook	2 (2)	Cavanaugh 2009 [54]; DeWalt 2006[55]
Monitoring devices	5 (5)	Bosworth 2018; 2009 [51, 52]; DeWalt 2012; 2006 [55, 56]; Lee 2017 [65]
Patient-own personality/psychological profile	1 (1)	Eikelenboom 2016 [64]
iPod	1 (1)	Carroll 2019 [53]
Other	5 (4)	Bosworth 2009 [51]; Kalichman 2014 [57]; Lee 2017 [65]; Murray 2007 [59]
**Patient knowledge/understanding/psychological support**		
Tailoring to individual: information and/or responses including encouragement, coaching, referrals, freephone numbers, and specific elements focusing on HL	16 (14)	Bosworth 2018; 2009; 2005 [50–52]; Carroll 2019 [53]; Cavanaugh 2009 [54]; DeWalt 2012; 2006 [55, 56]; Eikelenboom 2016[64]; Kalichman 2014 [57]; Kiser 2012 [58]; Lee 2017 [65]; Murray 2007 [59]; Negarandeh 2013[63]; Schillinger 2008;2009 [61, 62]
Understandable materials including language and/or culturally sensitive	13 (10)	Bosworth 2009[51]; Cavanaugh 2009 [54]; DeWalt 2012; 2006 [55, 56]; Kalichman 2014 [57]; Kiser 2012 [58]; Lee 2017 [65]; Murray 2007 [59]; Negarandeh 2013[63]; Schillinger 2008;2009 [61, 62]
Action plans, Goal setting	7 (6)	Bosworth 2018 [52]; DeWalt 2006 [55]; Eikelenboom 2016 [64]; Kalichman 2014 [57]; Lee 2017 [65]; Schillinger 2008;2009 [61, 62]
Memory aids incl. personalised prompt lists, adherence and mnemonic strategies, reminders and involvement of family/friends	6 (5)	Bosworth 2018; 2005 [50, 52]; Carroll 2019 [53]; Kalichman 2014 [57]; Murray 2007 [59]
Teach-back	5 (5)	DeWalt 2006 [55]; Kiser 2012 [58]; Lee 2017 [65]; Negarandeh 2013 [63]; Press 2016 [60]
Self-monitoring	4 (4)	Bosworth 2018; 2009 [51, 52]; Eikelenboom 2016[64]; Lee 2017 [65]
Motivational interviewing/enhancement	2 (2)	Bosworth 2018 [52]; Kalichman 2014 [57]
Role play	2 (2)	Carroll 2019 [53]; Kalichman 2014 [57]
Demonstration	2 (2)	Kiser 2012 [58]; Press 2016 [60]
Teach to goal	2 (2)	DeWalt 2012 [56]; Press 2016 [60]
Problem solving	2 (1)	Kalichman 2014 [57]
Brainstorming	1 (1)	DeWalt 2006 [55]
Patient profiling/screening	1 (1)	Eikelenboom 2016 [64]

### Work Package 3 (WP3): Qualitative investigation of the experiences of people living with MSK pain, HCPs and experts around self-management of MSK pain (objective 3)

#### Participant characteristics

Three focus groups with people with MSK pain from the community (CFG) and one focus group with professional stakeholders (PFG) were conducted, each lasting between 60–120 min. The three CFGs included a total of 17 participants (CFG 1 = 7, CFG 2 = 5, CFG 3 = 5) which comprised 13 women and 4 men with an mean age of 72.2 (SD 13.4) years and included a mix of race/ethnic backgrounds (white British = 12, south Asian = 4, white Canadian = 1). The PFG sample (*n* = 12) included a range of professional groups who are all directly involved in the care or support of people with MSK pain: 3 third sector workers, 2 GPs, 2 physiotherapists, 1 nurse, 1 occupational therapist, 1 pharmacist, 1 rheumatologist and 1 psychologist. The PFG comprised seven women and 1 man, with a wide range of experience levels (mean: 19 years providing care or support to people with MSK pain).

#### Key findings after thematic analysis

Two analytic categories were identified within the data from both people with MSK pain and the professional stakeholders. Within each theme, several sub-themes were also identified. These themes and sub-themes are described briefly below with illustrating quotes.

#### Theme 1: HCP education and training.

##### Understanding HL

Both groups highlighted the importance of understanding the concept of HL. In particular, that HCPs should be educated and trained to understand its importance and impact on health, specifically self-management, and consequently its relevance to health care practice and service provision.

*‘It’s education from the experts that we need isn’t it?’* (CFG)

*‘I think health literacy should be part of undergraduate medical and clinical training as a matter of course.’ *(PFG)

*‘Any intervention needs to be owned and understood by the person to whom it applies. And, in order for somebody to self-manage, they need to understand what the condition is, and what they can do about it, within their context.’* (PFG).

*‘If we come up with some sort of education, that would be helpful for the wider community in terms of giving them awareness, or giving them any kind of self-management programme to help them with their aches and pains and with their muscles and joints’.* (CFG).

##### Understanding MSK conditions in the context of HL

The psychosocial implications of MSK conditions with a particular focus on ageing, social construction of MSK, co-morbidities including mental health and access to services were deemed important when understanding MSK conditions in the context of HL.

*‘For some [patients], it will be about levels of health literacy. The problem with things like health literacy is, as I’ve said, everything needs to be holistic.’ *(PFG)

*‘It [health literacy] looks at everything from social to psychology to see what they’re picking up in that section. Are they picking up the social aspect? I find that so much more now about people’s pains, understanding and beliefs.’ *(PFG).

##### Understanding the complexity of health literacy at the level of individuals and communities

The training needs to address how patients may mask limited HL, and the importance of seeing the patient in the context of their personal, familial and social resources and support was felt to be crucial. HCP training also needs to enable them to acknowledge and understand what the patient/care-giver knows, including their health beliefs and expectations.

*‘They [doctors] generalise a lot of things and I quite agree with [X] as they (Drs) told [X] to take two times a day. The doctor says, ‘Take this three times a day,’ but they’re not really personally talking about you. They have to go deeper into what works’. *(CFG).

*‘The system is changing and it’s whether everybody in the community fully understands that actually, when it comes to illness, it’s more back on them to be in control of it. Whereas, actually some people just don’t want to be in control. They want to be told’.* (PFG).

##### Communication skills in working with patients with limited HL

Use of language and terminology was very important from the perspective of those with MSK pain. Suggestions of how to improve communication with patients to enable them to better retain information included alternative approaches e.g. teach-back, audio and visual aids etc. The importance of non-verbal communication, breaking bad news, and exploring patient health beliefs and expectations were also highlighted in the focus groups.

*‘Sometimes you just need somebody to go through, explain and be able to go a bit deeper with them rather than just being given the information.’ *(PFG).

*‘You go to a consultation with a dietician and when you walk out, how much can you retain? Not much, too much information’.* (CFG).

##### Understanding importance of timeliness of information and changing patient needs

Both groups agreed that training and education on identifying and responding to reactions of shock, anxiety, denial, guilt, blame is important. Providing timely information and prioritising information at different time points, whilst avoiding information overload, was deemed necessary when considering people with limited HL. Liaison with other providers and providing follow-up were also identified as important components for HCPs to consider.

*‘It’s also about the relative importance of musculoskeletal conditions compared to other things in terms of training, isn’t it? You only have to look at how diabetes, heart disease and some of these other conditions are prioritised. Of course, the other thing is that most of these people will have five or six other health conditions at the same time. Whilst the priority today might be aches and pains, the priority tomorrow will be diabetes, or blood pressure, or ten other things. I don’t think arthritis has that sense of priority for patients or for healthcare practitioners. We need to set this as priority.’* (PFG).

*‘They (HCPs) gave you too much information in one day for you to really take in. You can go back another time now and do it again’.* (CFG)

#### Theme 2: Patient and caregiver education and support.

##### Knowledge and understanding

To help patients understand their MSK pain the participants of the focus groups agreed that education and support is vital and should be condition-specific, accessible, reliable, and trustworthy. Specific knowledge and understanding of the side effects of analgesics, self-management strategies, sources of information, and relevant services including physiotherapy.

*‘I’m starting straight from the beginning of the consultation now. It’s massively changed my practice and I’m tending to not have to give out as much information leaflets and having more time to sit with people in clinic to sort of watch the video ‘Three minutes understanding pain’, especially with the younger chaps that don’t really open up.’* (PFG).

*‘There are a lot of things that we need to be guided into and educated well about, so then we can take the right steps.'* (CFG)

##### Modes of information delivery

The participants felt strongly that the way in which information is delivered to patients should be clear and accessible. Some of the suggestions from the groups included pamphlets, videos, pictures, audio clips, on YouTube, in GP surgeries, TV, physiotherapy clinics, and web-based resources; Regular text messages with prompts, updates and reminders were felt to be useful.

*‘…less information in a clearer format and signposting people as to where they can get more when they’re ready to receive it.'* (PFG)

*‘Don’t know if it could be something virtual, like webinars. I know people can drop in at times or there are videos they could watch and then people could start talking.’* (PFG)

*‘I'm talking about videos for treatment, I mean painkillers are great and this study is brilliant when you're getting different peoples, you know, I suppose, that’s one thing where it really would but to me it would be helpful if I could see somebody actually doing the exercises’.* (CFG).

##### Modes of support

The participants provided a range of perspectives on modes of support. Suggestions included a ‘floating’ advisor in GP surgeries to offer on-going support, integrated services, peer-support groups, expert-led support groups, community education sessions to raise awareness of MSK and self-management strategies, expert-moderated internet-based chat groups/forums and social media.

*‘Instead of leaving them and saying, ‘Right, off you go now,’ there’s a floating advisor you can just drop in. You may need to steer that person to that somebody and say, ‘Yeah, watch this video with them.’* (PFG).

*‘I think a group setting works quite well so like here we've shared ideas between each other, although there is a problem about possibly misinformation, you know, you can feed off other people and say, oh right, yes, well I never thought about that strategy or using a hot water bottle or doing this or doing the other and it may give you ideas that perhaps you hadn’t already thought about. So, meeting together in a group and perhaps have somebody come and show a video let’s say about strategies, I think that would be useful.’* (CFG).

### Work package 4a (WP4a): Triangulation of findings and a modified online Delphi consensus study

Triangulation of the findings resulted in the production of an evidence table. From this, 49 evidence statements across 5 themes (1. Training, support and materials for intervention deliverers, 2. Self-management support, 3. Education for patients, 4. Modes of information delivery, 5. Modes of support delivery) were created and used in the Delphi study (see Additional file 5).

A total of 23 participants participated in round 1 of the Delphi (16 women and 7 men, all identifying as white British), with a wide range of experience levels (average: 15 years proving care or support to people with MSK pain). The sample included different professional groups who are all directly involved in the care or support of people with MSK pain: 9 physiotherapists, 4 GPs, 2 third sector workers, 2 academic researchers, 1 nurse practitioner, 1 occupational therapist, 1 pharmacist, 1 rheumatologist, 1 psychologist and 1 Public Health specialist. The sample comprised of 5 individuals who stated they had expertise in HL.

**Fig. 3:**
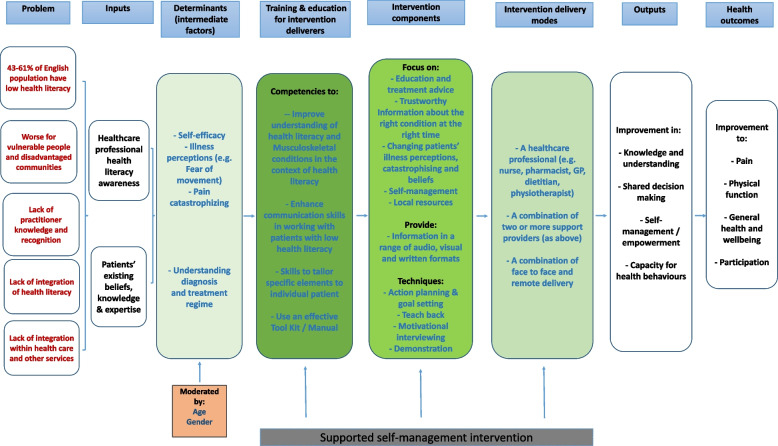
Evidence based Logic Model for Supported Self-Management Intervention Components for MSK Pain and Low HL

After analyzing the round 1 data, 12 evidence statements reached consensus and were removed from the second (final) round. The second round was completed by 21 (93.3%) participants from round 1 and a further 10 evidence statements reached consensus, which resulted in a total of 22 evidence statements relating to potential components and modes of delivery to include in the logic model (see Fig. 3).

These 22 evidence statements were then mapped back to the findings of the systematic review and assessed for the overall confidence in the evidence. This assessment found 8% of the statements demonstrated very low confidence, 23% demonstrated low confidence, 54% demonstrated moderate confidence and 15% demonstrated high confidence.

### Work package 4b (WP4b): Production of the logic model

Eight dimensions to the logic model were identified, each with their own domains: the problem, inputs, determinants, training and education (HCPs), intervention components, delivery modes, outputs and health outcomes (see Fig. 3). Key determinants identified included: self-efficacy, illness perceptions, and pain catastrophising. Several key intervention components were identified for example providing information in diverse formats (e.g. audio, video and written materials); action planning and goal setting; visual demonstrations of exercises. In terms of modes of delivery support the logic model highlights that it should be multi-professional using a combination of delivery modes including face to face and remote delivery.

## Discussion

Supported self-management interventions are widely used to improve the health of those with MSK pain [5]. Such interventions often have multiple interacting components and classed as complex interventions. In the United Kingdom, the Medical Research Council (MRC) is widely cited for its guidance on developing and evaluating complex interventions [66]. This study drew on this guidance and that of others such as O’Cathain et al. (2019) by using a mixed-method approach to produce a model for inclusive supported self-management intervention(s) for MSK pain that takes account of varying levels of HL [67].

The findings of work package 1 (secondary data analysis) demonstrated that low HL is associated with poorer (longitudinal) pain and function, which was independent in part from other baseline co-factors such as socio-demographic and health-related-factors. This provides further support to the findings of Lacey et al. (2018) who previously used the KAPS data alone to examine the impact of limited HL in a population with MSK pain [20]. However, these findings are in contrast with a systematic review by Loke et al. (2012), which did not find evidence of a clear relationship between limited HL and poorer functional outcomes in patients with chronic MSK conditions [3]. However, Loke et al. (2012) did highlight that many methodological weaknesses were evident within the studies identified and included in the review [3].

With regards to targets for intervention, a previous study of chronic pain clinics that served patients with low socioeconomic status found that lower levels of HL were associated with greater catastrophizing and lower pain-related self-efficacy [68]. A systematic review by Lowe et al. (2013) which examined the effectiveness of educational interventions in people with low literacy levels also demonstrated a modest effect on knowledge and self-efficacy, although there was a lack of high-quality evidence [4]. In work package 1 of this study, psychological factors including catastrophising, self-efficacy and illness perceptions were all found to be mediators of the relationship between poorer HL and greater pain and worse function longitudinally and should therefore be considered as targets for intervention in those with MSK pain. Consequently, these factors were included as targets for intervention in the logic model. There was little evidence of any clear and consistent moderation effects (of HL itself and of the mediation effect) from the analysed data, and hence moderation effects were not considered for the logic model.

The aim of work package 2 (systematic review) was not to estimate the overall effectiveness of self-management interventions as this has been addressed in several previous systematic reviews [69–71]. The aim was however, to focus on effective components of the interventions and the different modes of delivering supported self-management which may be suitable for those with limited HL. A large number of effective components were identified and these were grouped into 1. mode of delivery, 2. type of healthcare professional delivering the intervention, 3. training for intervention deliverers, 4. intervention materials and 5. patient knowledge, understanding and support.

The findings from work package 3 (focus groups) also identified a number of intervention components which were deemed important to HCPs and individuals with MSK pain. These components were grouped into 2 overarching themes, 1. HCP education and training, 2. Patient and caregiver education and support. Following the thematic analysis, it was clear that there was significant overlap between the effective components identified in work package 2 (systematic review) and the sub-themes identified in the focus group data (e.g. mode of delivery, training for HCPs and support for patients).

The triangulation of the data from work packages 2 and 3 grouped together the data sources and explored commonalities and divergence. The final evidence table produced from this process included a large number of intervention components and modes of delivery (*n* = 49) which were grouped into 1 of 5 themes (1. Training, support and materials for intervention deliverers, 2. Self-management support, 3. Education for patients, 4. Modes of information delivery, 5. Modes of support delivery).

Evidence statements were produced from the evidence table and were then considered by the stakeholders participating in the Delphi consensus study (work package 4). The majority (69%) of the evidence statements were associated with moderate or high levels of confidence in the evidence when risk of bias was assessed. The Delphi narrowed down the 49 evidence statements to 22 evidence statements, which the group felt should definitely be included in the logic model. At least one evidence statement was selected from each of the themes identified in the triangulation process.

### Strengths and limitations

To our knowledge, this is the first study to identify key components of supported self-management interventions suitable for individuals with MSK pain and varying levels of HL. We used a mixed methods approach, including both quantitative and qualitative methods. Conducting secondary data analysis of three large data sets was an efficient approach as it allowed us to access relevant data from 3 different studies with a collective large sample size.

Incorporating a systematic review enabled us to identify and synthesise all relevant evidence and assess our confidence in the components of interventions and modes of delivery selected for inclusion in the logic model. The focus groups and the Delphi study included a range of important stakeholders including people with MSK pain, multidisciplinary HCPs and third sector workers, therefore gained a wide range of relevant perspectives on what would be important to include in a supported self-management programme and what would be feasible to deliver within primary care. The team worked hard to involve seldom heard voices in the project and specifically approached groups in the community who may experience barriers to accessing services or are under-represented in healthcare decision making. However, there were some limitations surrounding the other samples recruited. For example, the professional focus group included only one male participant and all identified as white British. The participants in the Delphi study represented a better gender mix, but again there was only one participant that was not of white British origin.

Some findings that might have been expected to arise in the professional focus group, such as the ways in which promoting patient knowledge of the causes and mechanisms of pain in MSK conditions, were not brought up in the discussions. Factors such as this could be explored in future studies.

A key strength of this study was that PPIE was included at each stage of this research, from study conception through to analysis and agreeing the final logic model.

## Conclusions

This study has enabled us to produce a person-centered, evidenced-based logic model that describes evidence-based intervention targets, effective intervention components and modes of delivery to support self-management and explains how these can lead to improved health outcomes for patients with MSK pain and varying levels of HL.

The model is acceptable to both patients and HCPs and has potential for significant impact on the management of MSK pain and for improving patient health outcomes. The next steps are now to draw on this logic model to inform the future refinement and evaluation of new, inclusive supported self-management intervention(s) for MSK pain that can be delivered in primary care.

## Supplementary Information

**Additional file 1. **Details of datasets.

**Additional file 2. **OVID MEDLINE Search strategy.

**Additional file 3. **Case Vignette.

**Additional file 4. **Work package 3 data collection and analysis flowchart.

**Additional file 5. **Evidence statements for Delphi study.

**Additional file 6. **Estimates of association of baseline socio-demographic variables with follow up pain and function outcomes (and effect moderation with health literacy).

**Additional file 7. **Mediation and moderation of mediation of the association between inadequate health literacy and poor pain and function at follow up.

**Additional file 8. **Work package 2 TIDIER checklist.

## Data Availability

The datasets used and/or analysed during the current study are available from the corresponding author on reasonable request.

## Abbreviations

ARC: Applied Research Collaborations
CFG: Community focus group
EASIER: AchEs And painS and lIving bEtteR
HCPs: Healthcare Professionals
HL: Health literacy
KAPs: Keele Aches and Pains Study
LTCs: Long-term conditions
MRC: Medical Research Council
MSK: Musculoskeletal
NIHR: National Institute for Health and Care Research
PPIE: Patient and Public Involvement and Engagement
PFG: Professional focus group
RCT: Randomised controlled trial
STEMS: Stepping up the Evidence for Musculoskeletal Services
SEM: Structural equation modelling
TAPS: Treatment of Aches and PainS
TIDIER: Template for Intervention Description and Replication
WEA: Workers Education Association
WP: Work package
