# Supplementary material for: Supported self-management for all with musculoskeletal pain: an inclusive approach to intervention development: the EASIER study
Source: BMC Musculoskelet Disord. 2023 Jun 10;24:474. doi: 10.1186/s12891-023-06452-4 (PMC10257331; doi:10.1186/s12891-023-06452-4)
Supplement: Supplementary file 1 — Additional file 1. Details of datasets. [file 12891_2023_6452_MOESM1_ESM.docx]

**Additional file 1: Details of datasets**

**KAPS**

KAPS was a cohort study of adults aged ≥18 years who consulted for common MSK conditions in primary care, conducted in 14 general practices within Staffordshire and the West Midlands, UK. 4,720 patients were sent a baseline questionnaire after their consultation, and responders were followed-up at 2-months and 6-months. 1,890 (40.0%) patients returned a completed baseline questionnaire, with 75.8% responding at 2-month, and 78.7% responding at 6-month, follow-up.

**TAPS**

TAPS was a pilot cluster randomised controlled trial of adults aged ≥18 years who consulted for common MSK conditions (back pain, knee pain, neck pain, shoulder pain and/or multi-site pain) in primary care, carried out in 8 general practices within Staffordshire and the West Midlands, UK. 1237 patients were mailed a baseline questionnaire after their consultation, and responders were followed up monthly and at 6 months. 524 (42.4%) patients returned a completed baseline questionnaire and were recruited to the study with 518 (98.9%) responding to at least one monthly follow up and 479 (91.4%) at 6 months.

**STEMS**

STEMS was a pilot cluster randomised controlled trial (RCT) of adults aged ≥18 years with MSK problems, conducted in 4 general practices in one town with high deprivation in Cheshire, UK. 2,696 patients who consulted a general practice or physiotherapy services for MSK pain were mailed a baseline questionnaire, and followed-up at 2, 6 and 12 months. 978 patients (36%) returned a completed baseline questionnaire; response to follow-ups was 74.3% at 2 months, 78.4% at 6 months, and 70.7% at 12 months.
