# Supplementary material for: Supported self-management for all with musculoskeletal pain: an inclusive approach to intervention development: the EASIER study
Source: BMC Musculoskelet Disord. 2023 Jun 10;24:474. doi: 10.1186/s12891-023-06452-4 (PMC10257331; doi:10.1186/s12891-023-06452-4)
Supplement: Supplementary file 2 — Additional file 2. OVID MEDLINE Search strategy. [file 12891_2023_6452_MOESM2_ESM.docx]

**Additional file 2: OVID MEDLINE Search strategy**

For Ovid: The following table is an explanation of the symbols used in the search strategy below.

/ indicates an index term (MeSH/EMTREE heading)

exp before an index term indicates that all subheading were selected

.ti,ab,kf. indicates a search for a term in title/abstract/word(s) in keyword [MEDLINE]

$ at the end of a term indicates that this term has been truncated.

adj indicates a search for two terms where they appear adjacent to each another

adjn indicates a search for two terms where they appear within n words of each another

Searches

1 exp Health Literacy/

2 literacy/

3 ((literac$ or literate$ or illitera$) adj3 health).ti,ab,kf.

4 ((literal or literacy) adj (skill$ or problem$ or difficult$ or understand$)).ti,ab,kf.

5 Educational Status/

6 ((numerac$ or numerate$ or numerical or innumera$) adj3 health).ti,ab,kf.

7 ((numerical or numeracy) adj (skill$ or problem$ or difficult$ or understand$)).ti,ab,kf.

8 Reading/

9 (reading adj3 health).ti,ab,kf.

10 (reading adj (skill$ or problem$ or difficult$ or understand$)).ti,ab,kf.

11 Comprehension/

12 ((comprehension or comprehend$) adj3 health).ti,ab,kf.

13 ((comprehension or comprehend$) adj (skill$ or problem$ or difficult$ or

understand$)).ti,ab,kf.

14 newest vital sign.ti,ab,kf.

15 NVS.ti,ab,kf.

16 rapid estimate of adult literacy.ti,ab,kf.

17 REALM.ti,ab,kf.

18 TOFHLA.ti,ab,kf.

19 (education$ adj (status or level$ or attain$)).ti,ab,kf.

20 health communication/

21 health communication$.ti,ab,kf.

22 or/1-21

23 self care/

24 self management/

25 self medication/

26 self car$.ti,ab,kf.

27 self help.ti,ab,kf.

28 self guided.ti,ab,kf.

29 self directed.ti,ab,kf.

30 self manag$.ti,ab,kf.

31 (patient$ adj3 directed).ti,ab,kf.

32 (patient$ adj3 guided).ti,ab,kf.

33 (psychoeducation$ or psycho education$).ti,ab,kf.

34 self improvement.ti,ab,kf.

35 (patient$ adj3 educat$).ti,ab,kf.

36 (patient$ adj3 teach$).ti,ab,kf.

37 (patient$ adj3 train$).ti,ab,kf.

38 expert patient$.ti,ab,kf.

39 lay led.ti,ab,kf.

40 peer led.ti,ab,kf.

41 (patient$ adj3 (focus$ or participat$ or centr$ or center$ or empower$ or support$ or

collaborat$ or co-operat$ or cooperat$)).ti,ab,kf.

42 or/23-41

43 exp meta-analysis as topic/

44 meta-analysis/

45 meta-analysis.pt.

46 meta analysis.ti,ab,kf.

47 metaanalysis.ti,ab,kf.

48 systematic review.pt.

49 (systematic$ adj3 review$).ti,ab,kf.

50 (MEDLINE or Pubmed).ab.

51 (EMBASE or Cochrane).ab.

52 (PsycINFO or CINAHL$).ab.

53 prospero.ab.

54 prisma.ab.

55 systematic reviews.jn.

56 Cochrane database of systematic reviews.jn.

57 or/43-56

58 22 and 42 and 57

59 limit 58 to yr="2009 –Current”
