# Supplementary material for: Supported self-management for all with musculoskeletal pain: an inclusive approach to intervention development: the EASIER study
Source: BMC Musculoskelet Disord. 2023 Jun 10;24:474. doi: 10.1186/s12891-023-06452-4 (PMC10257331; doi:10.1186/s12891-023-06452-4)
Supplement: Supplementary file 3 — Additional file 3. Case Vignette. [file 12891_2023_6452_MOESM3_ESM.docx]

**Additional file 3: Case Vignette**

**Who is Robert?**

**
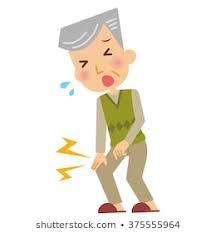
**

- 59 years old. Lives in Hanley with his 56 year old wife, Jasmine.
- Jasmine has breathing problems. She finds it difficult to manage stairs or to walk for more than 15 minutes without a rest.
- Two children – 3 grandchildren:
  - 31 year old son lives with wife and young child, two streets away. Son and daughter-in-law work full-time for Stoke Council.
  - 28 year old daughter lives with partner and two children on the same street. Both work full-time: daughter for Next in Hanley and partner is a self-employed electrician.
- Work: Robert and Jasmine run a small corner shop in the local area. The shop is open from 8 in the morning till 10 at night, 7 days a week.
- Jasmine has her son’s 4-year old and her daughter’s 3 year old for three mornings a week. She collects her daughter’s 6 year old from school on the other two days and looks after her until one of her parents comes home in the evening.

**What about Robert’s aches and pains?**

- Robert has been having pain in his knees for a couple of years
- More and more difficulty in bending, standing and walking about
- Pain getting worse
- After a couple of weeks not sleeping because of the pain, Robert went to see his GP
- Doesn’t like to go to the doctor’s
- Appointments with the GP are 10 minutes long
- The GP told him that his has arthritis in both knees - this was six weeks ago.

**How does Robert manage his aches and pains?**

- GP gave him a prescription for painkillers, and some information leaflets
- She told him to read through the leaflets when he got home as they had good advice on how he manage his arthritis and his pain
- She said to come back if it got any worse
- Told him to book an appointment at reception with the physiotherapist to help him with exercises
- Painkillers are not helping
- Pain is getting worse
- Robert has very low mood
- Robert has not booked an appointment with the physiotherapist
- Robert has not booked an appointment with his GP
