# Supplementary figures and images for: Supported self-management for all with musculoskeletal pain: an inclusive approach to intervention development: the EASIER study
Source: BMC Musculoskelet Disord. 2023 Jun 10;24:474. doi: 10.1186/s12891-023-06452-4 (PMC10257331; doi:10.1186/s12891-023-06452-4)

**Additional file 4: Work package 3 data collection and analysis flowchart**


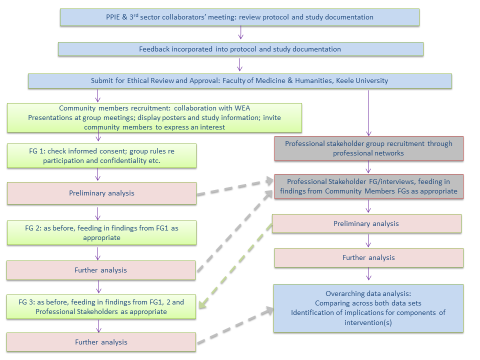

Supplement: Supplementary file 4 — Additional file 4. Work package 3 data collection and analysis flowchart. [file 12891_2023_6452_MOESM4_ESM.docx]
