# Supplementary material for: Supported self-management for all with musculoskeletal pain: an inclusive approach to intervention development: the EASIER study
Source: BMC Musculoskelet Disord. 2023 Jun 10;24:474. doi: 10.1186/s12891-023-06452-4 (PMC10257331; doi:10.1186/s12891-023-06452-4)
Supplement: Supplementary file 5 — Additional file 5. Evidence statements for Delphi study. [file 12891_2023_6452_MOESM5_ESM.docx]

**Additional file 5: Evidence statements for Delphi study**

**Online Delphi Survey**

**The following components of supported self-management interventions have been identified from previous research findings of the EASIER study (including reviews of published literature and focus group discussions and interviews with community people (with MSK pains), health care professionals and third sector collaborators). Please rank how strongly you feel they should be included within a suite of component and delivery options for a supported self-management intervention for people with musculoskeletal pain and varying levels of Health Literacy**

1. **Definitely include 2. Probably include 3. Probably not include 4. Definitely not include**

| **Themes** | **Statements** | **1** | **2** | **3** | **4** |
| --- | --- | --- | --- | --- | --- |
| **1. Training, support and materials for intervention deliverers (HCPs and others involved in delivery)** | | | | | |
| 1.1 | Intervention deliverer training and education on understanding health literacy and MSK conditions in the context of health literacy |  |  |  |  |
| 1.2 | Intervention deliverer training to enhance communication skills in working with patients with low health literacy |  |  |  |  |
| 1.3 | Intervention deliverer training to tailor specific elements to individual patient (e.g. focusing on health literacy, using cultural sensitive approach) |  |  |  |  |
| 1.4 | Intervention deliverer to receive ongoing support and training to ensure correct/effective intervention delivery |  |  |  |  |
| 1.5 | Intervention deliverer training to deal with changing patient needs and understanding their beliefs such as, anxiety, denial etc. |  |  |  |  |
| 1.6 | Intervention deliverer training to provide supported self-management to overcome stress and anxiety the MSK problem may be causing |  |  |  |  |
| 1.7 | **Intervention deliverer trainers to be skilled, or to receive necessary training, in educational techniques such as:** | | | | |
|  | **Teach back:** It is also called, ‘show me’ method where patients explain back or paraphrase the information given to them by the HCPs to check if they have understood correctly. |  |  |  |  |
|  | **Role play:** Able to act out or perform the part of a person or a character, for example as a technique in training |  |  |  |  |
|  | **Motivational interviewing:** It uses a directive, client centred and counselling style for stimulating and encouraging behaviour change of patients by exploring from them what may work best for them. |  |  |  |  |
|  | **Demonstration:** A practical explanation or exhibition of showing how something works or performed for example, showing a technique of exercise |  |  |  |  |
|  | **Teach to goal:** An educational intervention that teaches patients self-care skills until they reach patient defined learning goals / behavioural goals. |  |  |  |  |
| 1.8 | **The intervention deliverers should be provided with the resources that can be used to support/ guide them to deliver the intervention such as:** | | | | |
|  | **Tool Kit and Manual with pre-determined script**s (Example: manual to demonstrate back pain exercises and providing tips for supported self-management ) |  |  |  |  |
|  | **Computer software (e.g. for monitoring)** |  |  |  |  |
|  | **Understanding patients’ perceptions and beliefs** |  |  |  |  |
| 1. **Self-management support, materials/aids for patients** | | | | | |
| 2.1 |  |  |  |  |  |
|  | Educational Manual (e.g. manual to demonstrate exercises for back pain, information material regarding pain management tips and tricks) |  |  |  |  |
|  | Log book/ diaries (e.g. log book to record pain levels, writing reflective diaries) |  |  |  |  |
|  | Handouts with written instructions and images (e.g. exercise steps with diagrams) |  |  |  |  |
|  | Laminated cards with images (e.g. pictorial presentation of exercises) |  |  |  |  |
| 2.2  2.3 | **The intervention should use patient centred cognitive training techniques such as:** | | | | |
|  | Action planning and goal setting (realistic and achievable goals) |  |  |  |  |
|  | Problem solving skills (e.g. using brainstorming techniques to prioritise solutions) |  |  |  |  |
|  | **Use of monitoring aids:** | | | | |
|  | Monitoring devices e.g. Physical activity monitor (pedometer), Smart phone app to monitor use of and response to pain medication |  |  |  |  |
|  | Electronic devices (e.g. smartphone, tablet) pre-loaded with personal health record |  |  |  |  |
|  | Memory aids (incl. prompt lists, adherence and mnemonic strategies, reminders including involvement of family/friends) |  |  |  |  |
| **3. Education for patients** | | | | | |
| 3.1 | Receive condition specific information that is timely, accurate and appropriate whilst avoiding information overload |  |  |  |  |
| 3.2 | Build understanding of self-management strategies e.g. (Adjust medicine dosage, exercise etc.) |  |  |  |  |
| 3.3 | Receive information from reliable and trustworthy resources e.g. from trained HCPs or NHS website |  |  |  |  |
| 3.4 | Receive education and advice on treatments including pharmaceutical (e.g. pain medication (oral, topical)) and non-pharmaceutical options (e.g. exercise, physiotherapy, diet) |  |  |  |  |
| 3.5 | Raise awareness of locally available support groups for supported self-management (e.g. walking groups or exercise groups, disease-specific support groups) |  |  |  |  |
| **4. Modes of information delivery** | | | | | |
| 4.1 | Develop written information (e.g. information leaflets) clearly written in simple and understandable language keeping health literacy components in mind |  |  |  |  |
| 4.2 | Direct patients to reliable online information/ websites- such as, NHS, Versus Arthritis, Public Health England etc. |  |  |  |  |
| 4.3 | **Provide virtual modes of information delivery such as:** | | | | |
|  | Video clips (e.g. showing demonstration of back pain exercise) |  |  |  |  |
|  | Pictures (e.g. pictures showing self-managements techniques- such as hot and cold compression) |  |  |  |  |
|  | Audio clips/ Podcasts (e.g. verbal steps to follow exercises) |  |  |  |  |
| 4.4 | Set up regular text message service with prompts and updates |  |  |  |  |
| 4.5 | GP surgeries and physiotherapy clinics offering information delivery services such as community  support groups |  |  |  |  |
| 4.6 | Posting messages on social media e.g. Facebook, Instagram etc. |  |  |  |  |
| **5. Modes of support delivery** | | | | | |
| 5.1 | **Support should be delivered by:** | | | | |
|  | Face to face only |  |  |  |  |
|  | Remotely only |  |  |  |  |
|  | A combination of face to face and remote |  |  |  |  |
| 5.2 | **Support should be delivered by:** | | | | |
|  | A health care professional e.g. nurse, pharmacist, GP, dietitian, physiotherapist |  |  |  |  |
|  | A ‘floating advisor’ role in GP surgeries to offer on-going support (this can be non HCPs trained in MSK self-management interventions) |  |  |  |  |
|  | A community advisor e.g. educator (health, peer), counsellor, program coordinator |  |  |  |  |
|  | Peer-support groups |  |  |  |  |
|  | Expert patient led support groups |  |  |  |  |
|  | Community education sessions |  |  |  |  |
|  | Expert-moderated internet-based chat groups/forums |  |  |  |  |
|  | Social media support groups e.g. Facebook, Instagram |  |  |  |  |
| 5.3 | **Support should involve:** | | | | |
|  | A single mode (one of the above) |  |  |  |  |
|  | A combination of two or more modes |  |  |  |  |
