# Supplementary material for: Supported self-management for all with musculoskeletal pain: an inclusive approach to intervention development: the EASIER study
Source: BMC Musculoskelet Disord. 2023 Jun 10;24:474. doi: 10.1186/s12891-023-06452-4 (PMC10257331; doi:10.1186/s12891-023-06452-4)
Supplement: Supplementary file 6 — Additional file 6. Estimates of association of baseline socio-demographic variables with follow up pain and function outcomes (and effect moderation with health literacy). [file 12891_2023_6452_MOESM6_ESM.docx]

**Additional file 6. Estimates of association of baseline socio-demographic variables with follow up pain and function outcomes (and effect moderation with health literacy).**

| **KAPS** |  |  |  |  | **TAPS** |  |  | |  | |  | | **STEMS** |  |  |  |  |
| --- | --- | --- | --- | --- | --- | --- | --- | --- | --- | --- | --- | --- | --- | --- | --- | --- | --- |
|  | **PCS** | | **NRS-Pain** | |  | **MSK-HQ** | | | **NRS-Pain** | | | |  | **PCS** | | **BP** | |
|  | **B_1_** | **B_2_** | **B_1_** | **B_2_** |  | **B_1_** | | **B_2_** | | **B_1_** | | **B_2_** |  | **B_1_** | **B_2_** | **B_1_** | **B_2_** |
| Inadequate HL^#^ | -7.67^3^ | - | 1.96^3^ | - | Inadequate HL^#^ | -12.2^3^ | | - | | 2.22^3^ | | - | Inadequate HL^#^ | -5.67^3^ | - | -14.3^3^ | - |
|  |  |  |  |  |  |  | |  | |  | |  |  |  |  |  |  |
| *Baseline variables* |  |  |  |  | *Baseline variables* |  | |  | |  | |  | *Baseline variables* |  |  |  |  |
| Age (years)* | -1.55^3^ | 0.88 | 0.17^2^ | -0.32^1^ | Age (years)* | -0.36 | | 2.16 | | 0.08 | | -0.51 | Age (years)* | -1.76^3^ | -0.09 | -2.39^2^ | 1.45 |
| Gender (male) | 0.98 | -2.45 | -0.36^1^ | 0.62 | Gender (male) | 1.39 | | 0.64 | | 0.00 | | -0.52 | Gender (male) | 2.34^1^ | -0.76 | 5.80^2^ | -7.66^0^ |
| Age left school <=15 years | -4.17^3^ | 3.98^1^ | 1.06^3^ | -0.98^1^ | IMD_rank** | 0.24^2^ | | 0.19 | | -0.04^1^ | | -0.07 | IMD_rank** | 0.20^2^ | 0.29^2^ | 0.45^2^ | 0.56^0^ |
| No full-time education | -3.14^3^ | 6.87^2^ | 1.02^3^ | -0.65 | SOC_cat (3-4) | -0.15 | | -0.20 | | 0.07 | | -0.11 | No qualifications | -5.49^3^ | -0.12 | -10.8^3^ | -1.40 |
| No adult education | -3.16^3^ | 1.64 | 1.01^3^ | -0.32 | SOC_cat (5-9) | -3.40^1^ | | 4.66 | | 0.91^2^ | | 0.52 | Does not own home | -3.44^2^ | -1.37 | -9.17^3^ | -4.90 |
| Not employed | -6.78^3^ | -2.40 | 1.09^3^ | 0.37 | Live alone | -1.69 | | -2.51 | | 0.12 | | 0.91 | Not employed | -7.84^3^ | -0.08 | -14.4^3^ | 5.88 |
| MCS score* | 3.03^3^ | -1.38^0^ | -0.92^3^ | 0.08 | Not employed | -2.13^0^ | | 0.88 | | 0.56^1^ | | -0.77 | MCS score* | 2.77^3^ | -0.51 | 8.83^3^ | 0.70 |
| Comorbidity (>=2) | -7.64^3^ | 2.98 | 1.49^3^ | -0.56 | Comorbidity (>=2) | -5.12^3^ | | 1.53 | | 0.72^1^ | | 0.40 | Comorbidiy (>=2) | -8.63^3^ | -3.15 | -17.7^3^ | -3.84 |
| Duration (>3 months) | -7.19^3^ | 4.88^1^ | 2.04^3^ | -0.32 | TSK score* | -7.23^3^ | | -3.38 | | 1.20^3^ | | 0.78 | Duration (>3 months) | -6.76^3^ | -0.90 | -16.6^3^ | -5.07 |
| Baseline_NRS-Pain* | -24.3^3^ | 1.43^2^ | 7.54^3^ | -0.13 | Duration (>3 months) | -3.87^2^ | | -8.93^1^ | | 0.95^2^ | | 1.63 | Baseline_BP* | 2.65^3^ | -0.29 | 6.42^3^ | 0.01 |
| Baseline_PCS* | 8.50^3^ | -0.72 | -1.49^3^ | 0.39^0^ | Baseline_NRS-Pain* | -19.0^3^ | | 5.84 | | 4.66^3^ | | -3.76^0^ | Baseline_PCS* | 8.00^3^ | -0.12 | 15.7^3^ | -1.41 |
|  |  |  |  |  | Baseline_MSK-HQ* | 6.65^3^ | | 2.95^0^ | | -1.13^3^ | | -0.37 |  |  |  |  |  |

B_1_=Unadjusted unstandardised regression coefficient for the designated baseline variable against follow up pain or function outcome; B_2_=Unstandardised regression coefficient for the interaction term (product of the baseline variable and health literacy variable) against follow up pain or function outcome.

^#^HL: Adequate health literacy = never, rarely need help; Inadequate health literacy = sometimes, often, always need help.

Positive unstandardised regression coefficients designate improved function (in respect of PCS and MSK-HQ; higher PCS and MSK-HQ values denote greater function), reduced pain (in respect of BP; higher BP values denote less pain) and greater pain (in respect of NRS-Pain; higher NRS-Pain values denote increased pain). By contrast, negative unstandardised regression coefficients designate reduced function (in respect of PCS and MSK-HQ), greater pain (in respect of BP) and reduced pain (in respect of NRS-Pain).

Social Class (SOC-2010): 1 Managers, directors and senior officials; 2 Professional occupations; 3 Associate professional and technical occupations; 4 Administrative and secretarial occupations; 5 Skilled trades occupations; 6 Caring, leisure and other service occupations; 7 Sales and customer service occupations; 8 Process, plant and machine operatives; 9 Elementary occupations. SF12 = Short-Form_12 (MCS=Mental Component Scale, PCS=Physical Component Scale; BP=Body Pain; 0=worst health status, 100=best health status); NRS=Numerical Rating Scale (0-10 Pain scale; 0=no pain, 10=worst pain); HP-Behaviour Index = Health Professional Behaviour & Communication index: 0=Poor behaviour/communication, 72=Excellent behaviour/communication; TSK=Tampa Scale of Kinesiophobia (17-68 scale; 0=no fear, 68=most fear) (ref. ?); MSK-HQ=MuSKuloskeletal Health Questionnaire); IMD_rank=Index of Multiple Deprivation rank (1=most deprived rank, 32226=least deprived rank); TSK=Tamp Scale of Kinesiophobia (17=least fear, 64=most fear); MSK-HQ=Musculoskeletal Health Questionnaire score (0=most health and function problems, 56=least health and function problems).

* Baseline scale multiplied by 10 ** Baseline scale multiplied by 1000

^0^p<0.1, ^1^p<0.05, ^2^p<0.01, ^3^p<0.001
