# Supplementary material for: Supported self-management for all with musculoskeletal pain: an inclusive approach to intervention development: the EASIER study
Source: BMC Musculoskelet Disord. 2023 Jun 10;24:474. doi: 10.1186/s12891-023-06452-4 (PMC10257331; doi:10.1186/s12891-023-06452-4)
Supplement: Supplementary file 7 — Additional file 7. Mediation and moderation of mediation of the association between inadequate health literacy and poor pain and function at follow up. [file 12891_2023_6452_MOESM7_ESM.docx]

**Additional file 7: Mediation and moderation of mediation of the association between inadequate health literacy and poor pain and function at follow up.**

| ***Dataset***  **Outcome**  **> Mediator**  **# Moderator (of mediator)** | **Unstandardized (Standardized) regression coefficient** | **% Indirect effects (95% CI)** | **Standardized coefficients**  **Path_1: HL->Mediator \|\|**  **Path_2: Mediator->Outcome** |
| --- | --- | --- | --- |
|  |  |  |  |
| ***KAPS*** |  |  |  |
| Pain (0-10 NRS) at 6 months |  |  |  |
| > Catastrophising (0-36 scale) | 1.02 (0.126) | 57 (44, 69) |  |
| # Age (<65†, ≥65) |  |  | -0.060^3^ \|\| 0.027 |
| # Sex (female†, male) |  |  | 0.089^2^ \|\| 0.125 |
| # Co-morbidity (0†, ≥1) |  |  | 0.078^3^ \|\| -0.030 |
| # Full-time education (no†, yes) |  |  | 0.029^2^ \|\| 0.033 |
| # Baseline pain (<5†, ≥5) |  |  | 0.015 \|\| -0.047 |
| > Pain self-efficacy (0-60 scale) | 1.48 (0.185) | 83 (68, 97) |  |
| # Age (<65†, ≥65) |  |  | -0.034 \|\| 0.116^1^ |
| # Sex (female†, male) |  |  | 0.051 \|\| -0.135 |
| # Co-morbidity (0†, ≥1) |  |  | 0.034 \|\| -0.042 |
| # Full-time education (no†, yes) |  |  | 0.018 \|\| -0.124^1^ |
| # Baseline pain (<5†, ≥5) |  |  | -0.006 \|\| 0.063 |
| > Illness Perceptions (1-5 scale) | 0.82 (0.103) | 47 (34, 59) |  |
| # Age (<65†, ≥65) |  |  | -0.046^2^ \|\| -0.214^2^ |
| # Sex (female†, male) |  |  | 0.065^1^ \|\| -0.153 |
| # Co-morbidity (0†, ≥1) |  |  | -0.010 \|\| 0.123 |
| # Full-time education (no†, yes) |  |  | 0.009 \|\| 0.070 |
| # Baseline pain (<5†, ≥5) |  |  | -0.001 \|\| 0.100 |
| > Sleep problem (No, Yes) | 0.29 (0.036) | 16 (8, 23) |  |
| > Support {Emotional / Physical}  (No, Yes) | 0.03 (0.004) | 2 (0, 5) |  |
|  |  |  |  |
| Function (SF12-PCS, 0-100 scale) at 6 months |  |  |  |
| Catastrophising (0-36 scale) | -3.72 (-0.111) | 55 (42, 68) |  |
| # Age (<65†, ≥65) |  |  | 0.059^4^ \|\| 0.015^2^ |
| # Sex (female†, male) |  |  | -0.076^2^ \|\| -0.030 |
| # Co-morbidity (0†, ≥1) |  |  | -0.070^3^ \|\| 0.098 |
| # Full-time education (no†, yes) |  |  | -0.026^2^ \|\| -0.025 |
| # Baseline pain (<5†, ≥5) |  |  | -0.010 \|\| 0.124^2^ |
| Pain self-efficacy (0-60 scale) | -7.36 (-0.220) | 100 (87, 100) |  |
| # Age (<65†, ≥65) |  |  | 0.054^2^ \|\| -0.149^2^ |
| # Sex (female†, male) |  |  | -0.108^1^ \|\| 0.093 |
| # Co-morbidity (0†, ≥1) |  |  | -0.059 \|\| -0.076 |
| # Full-time education (no†, yes) |  |  | -0.022 \|\| 0.083 |
| # Baseline pain (<5†, ≥5) |  |  | 0.017 \|\| -0.143^2^ |
| Illness Perceptions (1-5 scale) | -3.31 (-0.100) | 48 (35, 61) |  |
| # Age (<65†, ≥65) |  |  | 0.043^2^ \|\| 0.167 |
| # Sex (female†, male) |  |  | -0.074^2^ \|\| 0.105 |
| # Co-morbidity (0†, ≥1) |  |  | 0.007 \|\| -0.129 |
| # Full-time education (no†, yes) |  |  | -0.007 \|\| -0.095 |
| # Baseline pain (<5†, ≥5) |  |  | 0.005 \|\| 0.017 |
| > Sleep problem (No, Yes) | -1.26 (-0.038) | 19 (10, 28) |  |
| > Support {Emotional / Physical}  (No, Yes) | -0.08 (-0.003) | 1 (0, 4) |  |
|  |  |  |  |
| ***TAPS*** |  |  |  |
| Pain (0-10 NRS) at 6 months |  |  |  |
| > Distress (0-10 scale) | 1.78 (0.159) | 88 (51, 100) |  |
| # Age (<65†, ≥65) |  |  | -0.095^1^ \|\| -0.050 |
| # Sex (female†, male) |  |  | 0.007 \|\| 0.001 |
| # Co-morbidity (0†, ≥1) |  |  | 0.034 \|\| 0.021 |
| # Social class (SOC2010 1-2†, 3-9) |  |  | -0.127 \|\| -0.039 |
| # Baseline pain (<5†, ≥5) |  |  | -0.033 \|\| 0.002 |
| > Confidence (0-10 scale) | 0.65 (0.058) | 33 (10, 57) |  |
| # Age (<65†, ≥65) |  |  | -0.023 \|\| -0.071 |
| # Sex (female†, male) |  |  | 0.035 \|\| -0.022 |
| # Co-morbidity (0†, ≥1) |  |  | -0.052 \|\| 0.074 |
| # Social class (SOC2010 1-2†, 3-9) |  |  | -0.017 \|\| 0.149 |
| # Baseline pain (<5†, ≥5) |  |  | -0.041 \|\| -0.020 |
| > HP-Behaviour (0-72 scale) | 0.04 (0.004) | 2 (0, 11) |  |
|  |  |  |  |
| Function (MSK-HQ, 0-56 scale) at 6 months |  |  |  |
| > Distress (0-10 scale) | -6.84 (-0.150) | 61 (33, 88) |  |
| # Age (<65†, ≥65) |  |  | 0.110^2^ \|\| 0.052 |
| # Sex (female†, male) |  |  | -0.023 \|\| 0.049 |
| # Co-morbidity (0†, ≥1) |  |  | -0.017 \|\| -0.065 |
| # Social class (SOC2010 1-2†, 3-9) |  |  | 0.094 \|\| -0.014 |
| # Baseline pain (<5†, ≥5) |  |  | 0.039 \|\| 0.027 |
| > Confidence (0-10 scale) | -3.28 (-0.072) | 30 (10, 50) |  |
| # Age (<65†, ≥65) |  |  | 0.024 \|\| -0.056 |
| # Sex (female†, male) |  |  | -0.053 \|\| -0.076 |
| # Co-morbidity (0†, ≥1) |  |  | 0.063 \|\| 0.030 |
| # Social class (SOC2010 1-2†, 3-9) |  |  | 0.038 \|\| -0.110 |
| # Baseline pain (<5†, ≥5) |  |  | 0.044 \|\| 0.035 |
| > HP-Behaviour (0-72 scale) | -0.22 (-0.005) | 2 (0, 12) |  |
|  |  |  |  |
| ***STEMS*** |  |  |  |
| Pain (SF12-BP, 0-100 scale) at 12 months |  |  |  |
| > Pain self-efficacy (0-60 scale) | -8.16 (-0.141) | 79 (47, 100) |  |
| # Age (<65†, ≥65) |  |  | 0.014 \|\| -0.096 |
| # Sex (female†, male) |  |  | -0.080 \|\| 0.012 |
| # Co-morbidity (≤1†, ≥2) |  |  | -0.059 \|\| -0.118 |
| # Education (no qualifications†,  qualifications) |  |  | 0.000 \|\| 0.226^2^ |
| # Baseline SF12-BP (<40†, ≥40) |  |  | 0.024 \|\| 0.210 |
| > GPAQ (Understanding)  (0-100 scale) | 0.07 (0.001) | 0 (0, 2) |  |
| > GPAQ (Experience) (0-100 scale) | -0.01 (0.000) | 0 (0, 7) |  |
|  |  |  |  |
| Function (SF12-PCS, 0-100 scale) at 12 months |  |  |  |
| > Pain self-efficacy | -3.45 (-0.140) | 95 (56, 100) |  |
| # Age (<65†, ≥65) |  |  | 0.023 \|\| -0.008 |
| # Sex (female†, male) |  |  | -0.081 \|\| -0.110 |
| # Co-morbidity (≤1†, ≥2) |  |  | -0.067 \|\| 0.043 |
| # Education (no qualifications†,  qualifications) |  |  | -0.003 \|\| 0.167 |
| # Baseline SF12-BP (<40†, ≥40) |  |  | 0.026 \|\| 0.219 |
| > GPAQ (Understanding)  (0-100 scale) | 0.07 (0.001) | 0 (0, 2) |  |
| > GPAQ (Experience) (0-100 scale) | -0.01 (0.000) | 0 (0, 7) |  |
|  |  |  |  |

Catastrophising scale (0=no catastrophising, 52=most catastrophising, ref Sullivan MJL et al. Physiol Assess 1995); Pain Self-Efficacy scale (0=least self-efficacy, 60=most self-efficacy, ref Nicholas MK Eur J Pain 2007); Brief Illness-Perception scale (1=least illness-perceptions, 5=highest illness perceptions, Weinman J et al. Psychology & Health 1996); Distress scale (0=least distress, 10=most distress); Confidence scale (0-10 scale, 0=least confidence, 10=most confidence); SF12 = Short-Form_12 (PCS=Physical Component Scale, BP=Bodily Pain; 0=worst health status, 100=best health status, ref. Ware and Gandek J Clin Epidemiol 1998); NRS=Numerical Rating Scale (0=no pain, 10=worst pain); HP-Behaviour Index = Health Professional Behaviour & Communication index (0=Poor behaviour/communication, 72=Excellent behaviour/communication, ref Holt and Pincus 2016); MSK-HQ=MuSKuloskeletal Health Questionnaire (0=most MSK problems, 56=least MSK problems, ref Hill JC BMJ Open 2016); GPAQ (General Practice Assessment Questionnaire, 0=little assessment, 100=most assessment, ref Mead N et al. BMC Family Practice2008)

% Indirect effect constrained to 0-100%. CI = Confidence Interval. ^1^p<0.05, ^2^p<0.01, ^3^p<0.001.

† reference (moderator) subgroup category for moderation of mediation analysis.
